# Increasing the Complexity in the MIL‐53 Structure: The Combination of the Mixed‐Metal and the Mixed‐Linker Concepts

**DOI:** 10.1002/chem.202003304

**Published:** 2020-12-14

**Authors:** Johannes Bitzer, Milada Teubnerová, Wolfgang Kleist

**Affiliations:** ^1^ Faculty of Chemistry and Biochemistry Industrial Chemistry—Nanostructured Catalyst Materials Ruhr University Bochum Universitätsstraße 150 44801 Bochum Germany

**Keywords:** breathing behavior, isoreticular, metal–organic frameworks, mixed-metal/mixed-linker, physisorption

## Abstract

The isoreticular mixed‐component concept is a promising approach to tailor the material properties of metal–organic frameworks. While isoreticular mixed‐metal or mixed‐linker materials are commonly synthesized, the combination of both concepts for the development of isoreticular materials featuring both two metals and two linkers is still rarely investigated. Herein, we present the development of mixed‐metal/mixed‐linker MIL‐53 materials that contain different metal combinations (Al/Sc, Al/V, Al/Cr, Al/Fe) and different linker ratios (terephthalate/2‐aminoterephthalate). The possibility of changing the metal combination and the linker ratio independently from each other enables a large variety of modifications. A thorough characterization (PXRD, ATR‐IR, TGA, ^1^H NMR, ICP‐OES) confirmed that all components were incorporated into the framework structure with a statistical distribution. Nitrogen physisorption measurements showed that the breathing behavior can be tailored by adjusting the linker ratio for all metal combinations. All materials were successfully used for post‐synthetic modification reactions with maleic anhydride.

## Graphical abstract

**Different metal combinations** and different linker ratios have been used to synthesize isoreticular mixed‐metal/mixed‐linker MIL‐53 materials. The direct synthesis procedure at ambient pressure resulted in a statistical distribution of all components within the framework structure. The material properties, particularly the breathing behavior, can be tailored by the selection of suitable combinations and ratios of the components.

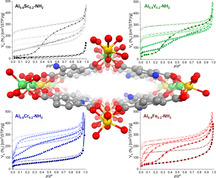

## Introduction

Multicomponent metal–organic framework (MOF) materials are gaining growing attention in this decade.^[1]^ The toolbox‐like design enables a high variability of components and, thus, various functionalities, often in combination with high porosity. Within this field, the synthesis of multicomponent metal–organic framework materials, in which several comparatively small and structurally simple building blocks are combined into one framework structure, is one of the most promising concepts.^[2]^

**Figure 1:**
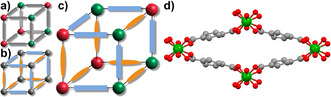
Models of isoreticular a) mixed‐metal, b) mixed‐linker and c) mixed‐metal/mixed‐linker metal–organic frameworks and d) a schematic representation of the diamond‐shaped pores of the MIL‐53 structure in the np‐form.

The advantage of isoreticular mixed‐component metal–organic framework materials, in which the metals or linkers are distributed over crystallographically equivalent lattice positions (Figure 1 a, b), is that the given framework structure can be retained while different properties thereof can be altered.[^2d^, ^2g^, ^3^] This way, linker molecules can be replaced by another type with similar geometry, but different functionality; this enables the development of a large library of materials from easily available and cheap starting materials. To date, the majority of publications on isoreticular mixed‐component metal–organic frameworks used either the mixed‐metal or the mixed‐linker concept.[^2d^, ^2g^, ^3c^, ^3d^, ^3e^, ^3f^] Only a few publications combined both concepts for the synthesis of isoreticular mixed‐metal/mixed‐linker metal–organic framework materials (Figure 1 c).^[4]^ However, only if such a combination of these two concepts is possible, all benefits of the toolbox‐like design can truly be exploited to tailor the properties of metal–organic frameworks. For a selected framework structure, the number of possible modifications exponentiates if the mixed‐metal and the mixed‐linker concepts are combined, even for a small number of available components.

Different one‐step or multi‐step synthesis procedures have been reported for the synthesis of isoreticular mixed‐component materials.[^3c^, ^5^] One‐step or direct methods are usually the most convenient ones. All starting materials are mixed during the synthesis and the resulting product is purified afterwards. Multistep methods usually use either a partial post‐synthetic modification or post‐synthetic exchange reactions for the synthesis of mixed‐component materials.^[3c]^ While the control over the whole process is much better in a multi‐step method, the effort is often significantly higher due to several synthesis and purifications steps. Furthermore, the formation of core–shell architectures has often been observed for these multi‐step methods, while a statistical distribution of linkers is frequently found in one‐step procedures.^[3c]^

The family of MIL‐53 materials is well‐investigated and has been synthesized with a variety of different metals and linker molecules.^[6]^ In the MIL‐53 structure (Figure 1 d), the trivalent metals have an octahedral coordination sphere with four oxygen atoms from linker molecules and two oxygen atoms from bridging μ^2^‐OH ions that are connecting neighboring metal centers. The resulting infinite M‐OH chains are connected by terephthalate linker molecules resulting in one‐dimensional diamond‐shaped channels. The vanadium analogue was named MIL‐47 and contains vanadium centers in the 4+ oxidation state and O^2−^ as the bridging moieties. Various mixed‐metal[^6^, ^7^] or mixed‐linker^[8]^ materials with MIL‐53 structure have been reported, which were shown to have tunable properties depending on the ratio of the incorporated metals or linkers.

In the present contribution, the MIL‐53 topology has been used for the formation of previously unknown mixed‐metal/mixed‐linker MIL‐53 materials, in which the respective components have been incorporated in a statistically distributed fashion into the framework structure. Various metal combinations (Al/Sc, Al/V, Al/Cr, Al/Fe) have been successfully used in combination with a mixture of terephthalate and 2‐aminoterephthalate as linker molecules. Furthermore, all materials were used for post‐synthetic modifications of the amine groups with maleic anhydride.

## Results and Discussion

**Scheme 1:**
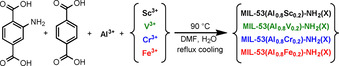
Schematic representation of the synthesis of MIL‐53(Al_0.8_M_0.2_)‐NH_2_(X) materials (M=Sc, V, Cr, Fe).

The development of a direct synthesis method for the herein presented mixed‐metal/mixed‐linker MIL‐53 materials seemed to be desirable to achieve the targeted statistical distribution of linkers and metals with a low effort. The previously developed direct synthesis procedures for MIL‐53(Al_0.8_M_0.2_)‐NH_2_ materials (M=Sc, V, Cr or Fe) were used for the synthesis of mixed‐metal/mixed‐linker materials (Scheme 1).^[7a]^ For each of the metal combinations, different ratios of terephthalate/2‐aminoterephthalate were used during the synthesis without any other changes to the procedures. The obtained MIL‐53(Al_0.8_M_0.2_)‐NH_2_(X) materials were labeled according to the percentage of 2‐aminoterephthalate with respect to the total amount of linkers. The ratios of incorporated terephthalate and 2‐aminoterephthalate linkers were determined by using liquid‐phase ^1^H NMR spectroscopy of digested samples. The found ratios were close to the expected values for all MIL‐53(Al_0.8_M_0.2_)‐NH_2_(X) materials (Table S1 in the Supporting information).

**Figure 2:**
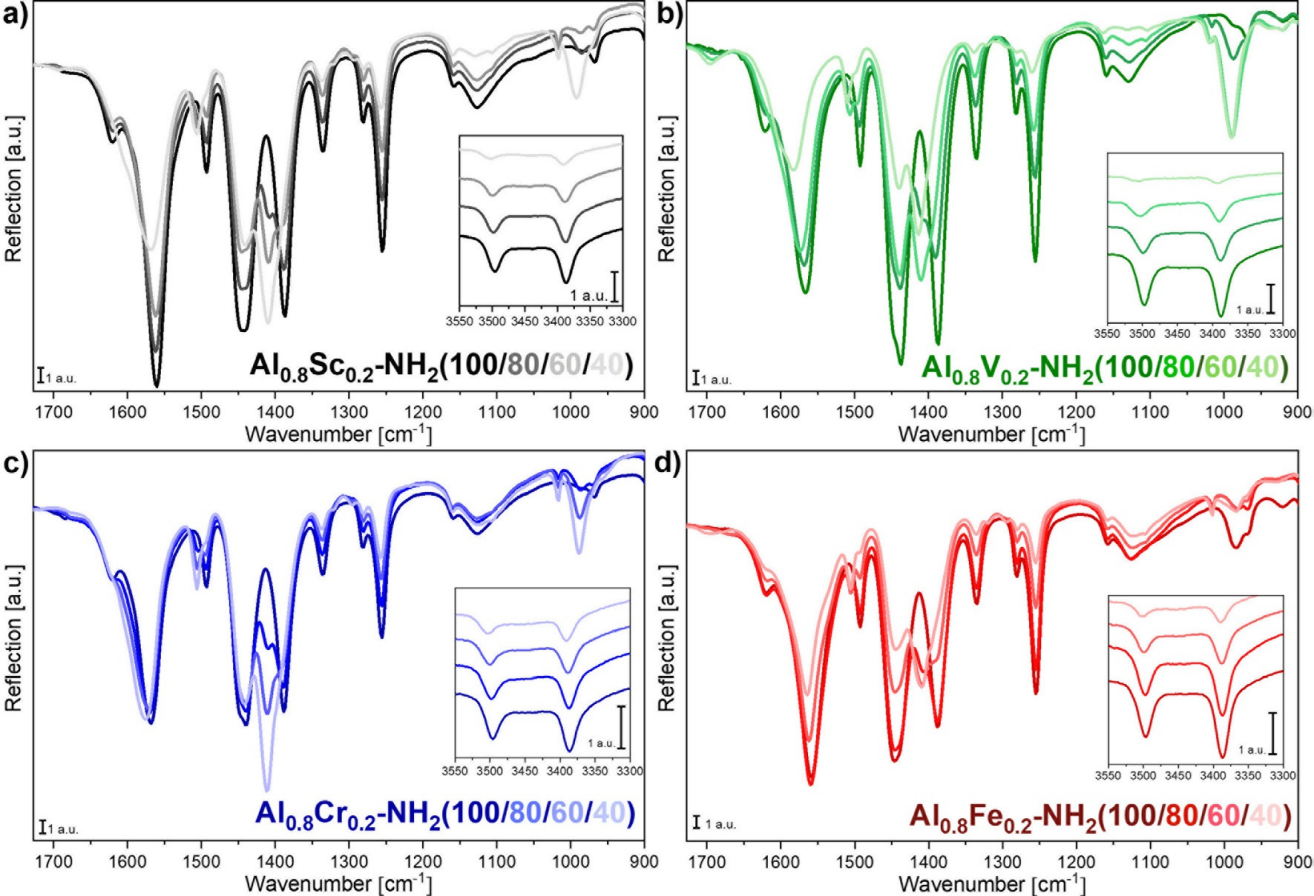
ATR‐IR spectra of MIL‐53(Al_0.8_M_0.2_)‐NH_2_(X) materials. The substitution of terephthalate for 2‐aminoterephthalate could be confirmed qualitatively based on the increasing and decreasing band intensities of the corresponding benzene ring vibrations of both linkers and the amine vibration of 2‐aminoterephthalate.

Recorded ATR‐IR spectra of all MIL‐53(Al_0.8_M_0.2_)‐NH_2_(X) materials (Figure 2) showed that no or only small amounts of residual linker molecules (1690–1675 cm^−1^) or *N*,*N*‐dimethylformamide (DMF, 1675–1665 cm^−1^) were present within the pores of the MIL‐53 structure. However, for MIL‐53(Al_0.8_V_0.2_)‐NH_2_(X) materials, this state was only achieved after additional suspension in DMF at 90 °C and a subsequent filtration and washing process (see the Experimental Section), since bands of residual linker molecules were observed in the IR spectra directly after the synthesis (Figure S2). The presence of various terephthalate/2‐aminoterephthalate ratios was visible from the changing intensities of several bands. With increasing amount of the incorporated terephthalate linker, the bands centered at 1387, 1492 and 1620 cm^−1^ decreased and the bands at 1410 and 1507 cm^−1^ increased in intensity. Furthermore, the intensity of the bands of amine vibrations (3497 and 3387 cm^−1^) decreased with decreasing 2‐aminoterephthalate content. Although quantitative statements based on the IR spectra are difficult, these observed trends were in accordance with the determined linker ratios based on ^1^H NMR measurements.

The band centered at 1125 cm^−1^, which could be assigned to the *δ(*OH) vibration of the μ^2^‐OH group interacting with water molecules within the pores,^[9]^ was similar for all materials within the series of Cr and Fe. However, the band intensity constantly decreased for V‐ and Sc‐containing materials with an increasing amount of terephthalate in the framework. Furthermore, a band close to 990 cm^−1^, which has previously been ascribed to the *δ(*OH) vibration of the μ^2^‐OH group without any interaction of solvent molecules,^[9]^ increased in intensity with increasing amount of terephthalate for all materials. Based on the currently available set of data, a conclusive explanation for this observation could not be provided.

The metal ratios of all MIL‐53(Al_0.8_M_0.2_)‐NH_2_(X) materials were determined using inductively coupled plasma optical emission spectroscopy (ICP‐OES; Table S2). For iron and chromium, the ratios were close to the expected values, which were present during the synthesis. In the case of vanadium, slightly higher aluminum contents and, for scandium, slightly lower aluminum contents than expected were found. Overall, the different linker ratios did not have any influence on the incorporated metal ratios compared to the materials containing only 2‐aminoterephthalate as linker.

**Figure 3:**
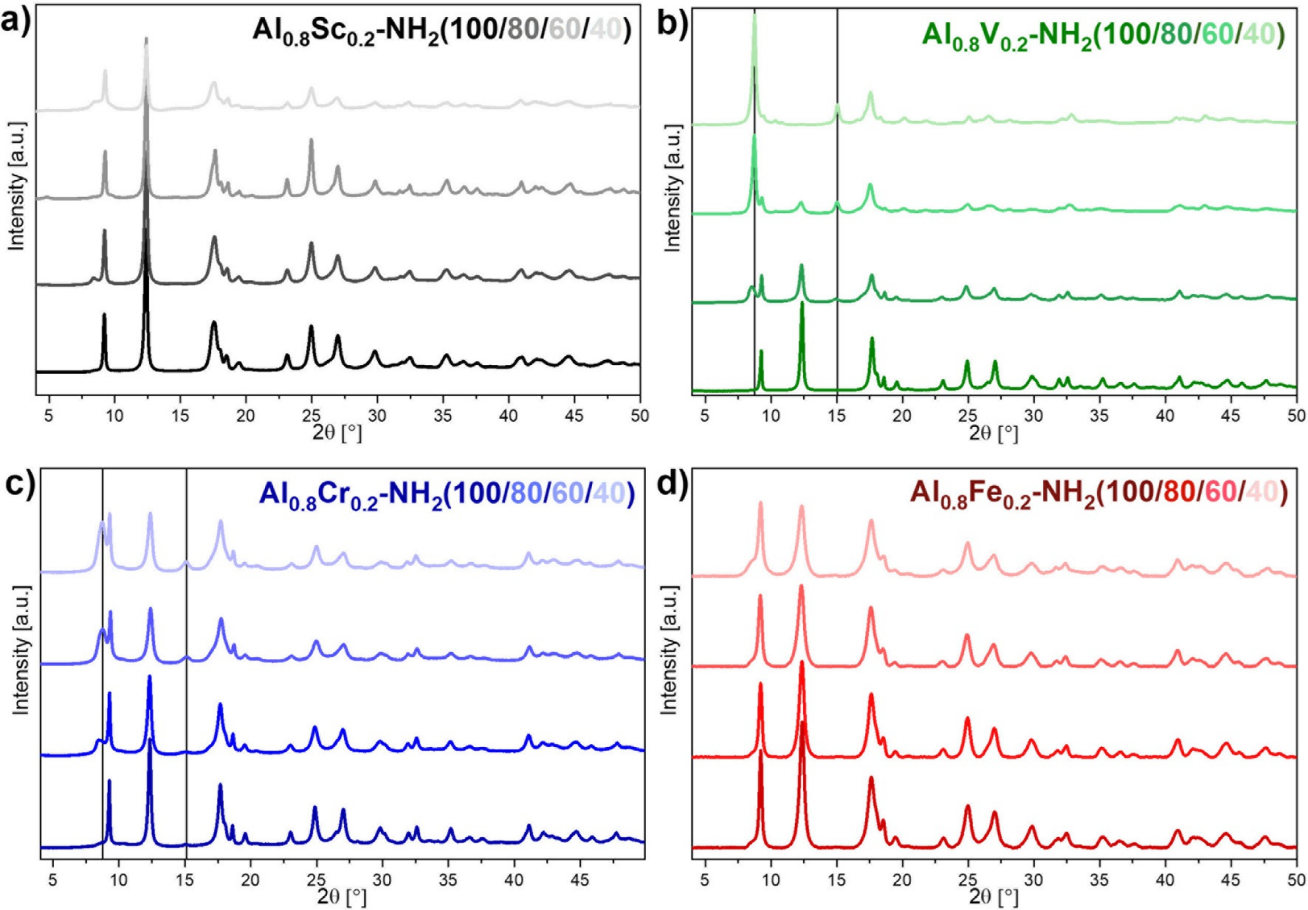
Powder X‐ray diffraction patterns of MIL‐53(Al_0.8_M_0.2_)‐NH_2_(X) materials showing typical reflections for the MIL‐53 structure in the np‐form and, for higher amounts of terephthalate, also the lp‐form. Characteristic reflections of the lp‐form are indicated by vertical lines.

Powder X‐ray diffraction patterns (Figure 3) showed that crystalline materials were obtained for all metal and linker combinations. The observed diffraction patterns for scandium‐ and iron‐containing materials were similar to reported patterns of MIL‐53(Al)‐NH_2_ in the narrow pore (np) form^[10]^ and showed only few changes upon the variation of the linker ratios, which was similar to previously reported mixed‐linker MIL‐53(Al)‐NH_2_(X).[^8a^, ^11^] Thus, the MIL‐53 structure was successfully obtained for all of these materials. For MIL‐53(Al_0.8_Cr_0.2_)‐NH_2_(X) materials, the reflections of the MIL‐53(Al)‐NH_2_ np‐form dominated the diffraction patterns for all linker ratios. However, reflections of presumably a large pore (lp) form (2*θ*=8.7°, 15.0°) increased in intensity with increasing content of terephthalate in the framework. This observation indicated that an increasing proportion of the particles featured a lp‐state. In the case of the vanadium‐containing materials, reflections corresponding to np‐ and lp‐forms were also visible for the mixed‐linker materials, but in contrast to the chromium‐containing materials, the changes were more pronounced. The np‐form was dominating for MIL‐53(Al_0.8_V_0.2_)‐NH_2_(80), whereas the lp‐form dominated for MIL‐53(Al_0.8_V_0.2_)‐NH_2_(60). The reflections of the np‐state were only hardly visible for MIL‐53(Al_0.8_V_0.2_)‐NH_2_(40) and the obtained reflection pattern was similar to the one observed for MIL‐47(V).^[12]^ Therefore, the lp‐form seemed to be favored already at comparatively low terephthalate contents. Thus, the incorporation of terephthalic acid into the MIL‐53‐NH_2_ structure had the strongest influence for vanadium‐containing materials, whereas only small changes were observed for chromium, scandium and iron.

Pawley refinements of the measured powder X‐ray diffraction patterns were performed to determine the unit cell parameters of the observed np‐ and lp‐phases starting from reported values (Table S3). The incorporation of terephthalate resulted in only small changes of the unit cell parameters for the corresponding phases.

**Figure 4:**
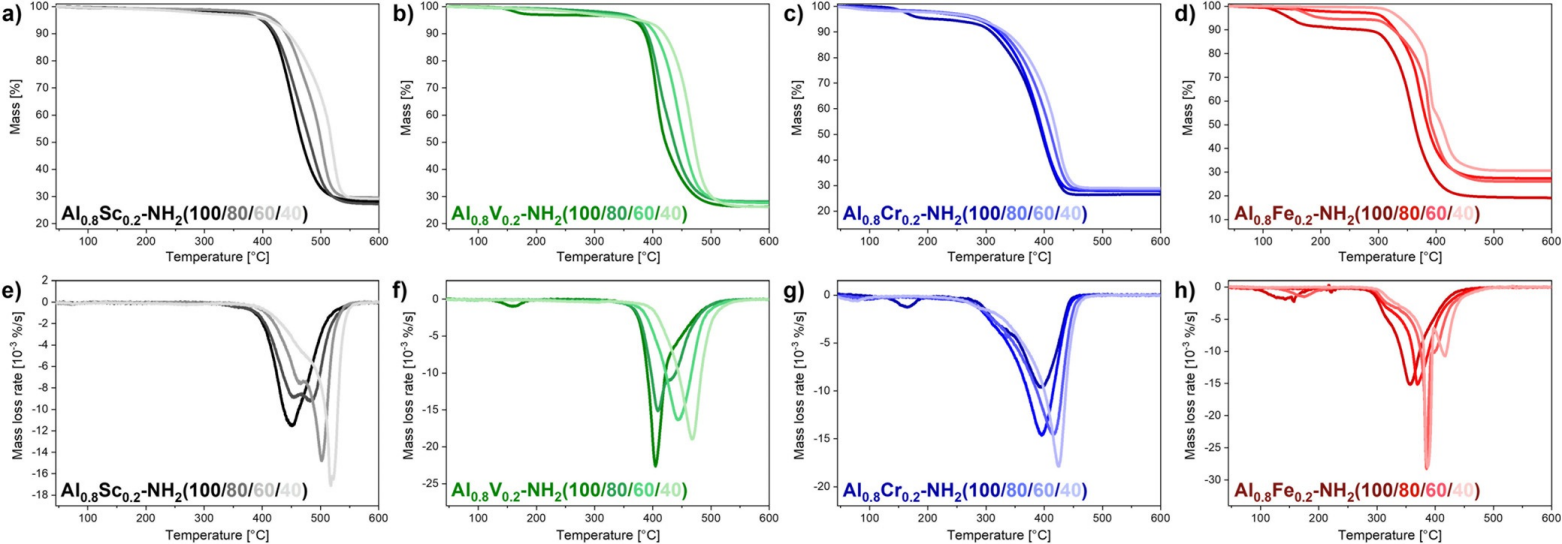
Results of the thermogravimetric analysis (a–d) and the corresponding first derivatives of the TG curves (e–h) of MIL‐53(Al_0.8_M_0.2_)‐NH_2_(X) materials performed in synthetic air. The thermal stability of these materials increased with an increasing amount of terephthalate in the frameworks for all metal combinations.

The thermal stability of monometallic mixed‐linker MIL‐53(Al)‐NH_2_(X) materials was previously shown to increase with an increasing amount of terephthalate and a decreasing amount of 2‐aminoterephthalate.^[8a]^ This trend was also found for the MIL‐53(Al_0.8_M_0.2_)‐NH_2_(X) materials (Figure 4). However, the differences of the stabilities depended on the metal combinations. Comparatively large shifts of the maximum decomposition rate to higher temperatures were observed for the MIL‐53(Al_0.8_Sc_0.2_)‐NH_2_(X) (450–520 °C) and the MIL‐53(Al_0.8_V_0.2_)‐NH_2_(X) (400–470 °C) series, while medium shifts were visible for the MIL‐53(Al_0.8_Cr_0.2_)‐NH_2_(X) (395–420 °C) an MIL‐53(Al_0.8_Fe_0.2_)‐NH_2_(X) (360–390 °C) series. The onset of the major decomposition step shifted in similar extents. Shoulders or additional small local minima were observed for several of these materials in the derivatives of the TG curves. However, these features also shifted depending on the linker ratios. In total, the continuous shift of the thermal stability corroborated the above claimed statistical incorporation of all components into the MIL‐53 structure. All materials showed a good stability up to 250 °C and can, therefore, also be potentially used for gas phase applications at elevated temperatures.

**Figure 5:**
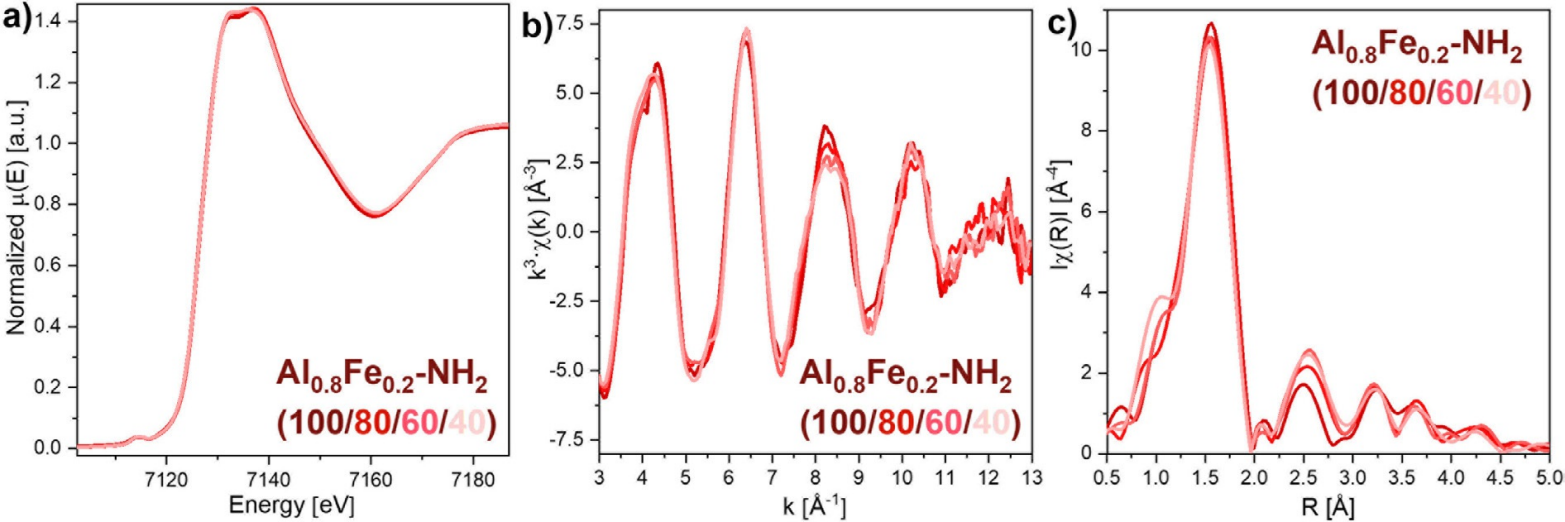
a) XANES, b) EXAFS and c) Fourier‐transformed EXAFS spectra of MIL‐53(Al_0.8_Fe_0.2_)‐NH_2_(X) materials recorded at the Fe K‐edge. The incorporation of terephthalate did not have any influence on the oxidation state of iron and had only a small influence on the local environment.

X‐ray absorption spectra (Figure 5) were recorded for the MIL‐53(Al_0.8_Fe_0.2_)‐NH_2_(X) materials to investigate whether the incorporation of terephthalate had an influence on the local chemical environment of the iron centers. The results confirmed that the oxidation state of the iron centers remained unchanged (+III) for all linker ratios. Apart from that, only small changes in the k‐ and R‐space were visible.

**Figure 6:**
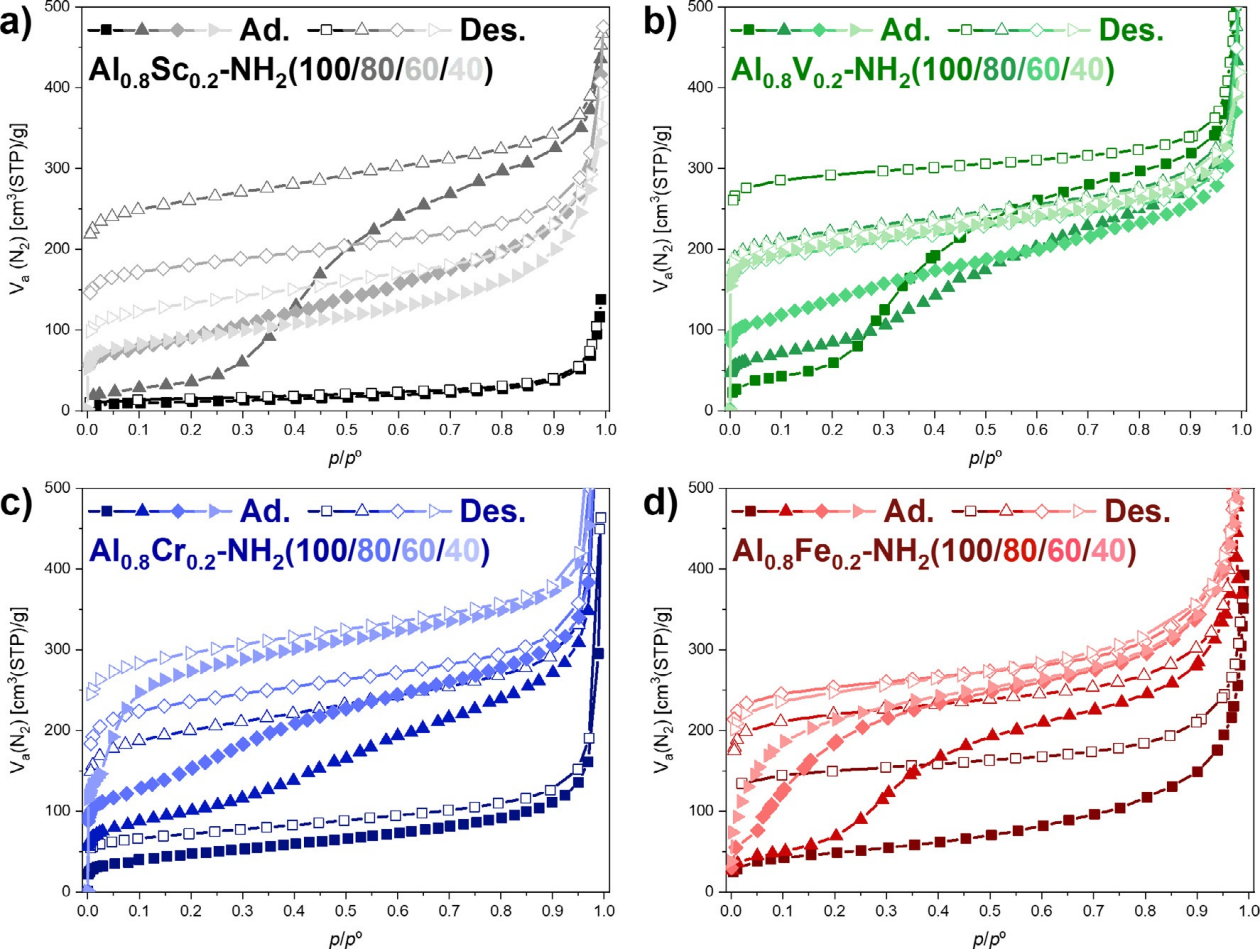
Nitrogen physisorption isotherms of MIL‐53(Al_0.8_M_0.2_)‐NH_2_(X) materials recorded at 77 K. The incorporation of terephthalate resulted in significant changes of the adsorption isotherms and, thus, in the breathing behavior of the MIL‐53 structures during nitrogen physisorption.

Nitrogen physisorption measurements have been performed to investigate the changes of the breathing behavior, which were induced by applying the mixed‐linker concept to the mixed‐metal MIL‐53(Al_0.8_M_0.2_)‐NH_2_ materials. The resulting isotherms showed several interesting features (Figure 6). Both, MIL‐53(Al_0.8_Cr_0.2_)‐NH_2_(100) and MIL‐53(Al_0.8_Fe_0.2_)‐NH_2_(100) showed isotherms with a significant difference between the adsorption and the desorption branches (Figure 6 c, d). As reported earlier,^[7a]^ we proposed that a breathing transition occurred only at high relative pressures, which would explain the missing S‐shaped adsorption isotherm that is usually related to a breathing behavior and the difference of the adsorbed volume between the adsorption and desorption branches.

The incorporation of different amounts of terephthalate in addition to 2‐aminoterephthalate resulted in significantly larger adsorbed volumes in the adsorption and the desorption branches of the MIL‐53(Al_0.8_Cr_0.2_)‐NH_2_(X) and MIL‐53(Al_0.8_Fe_0.2_)‐NH_2_(X) materials. The adsorbed volumes increased with an increasing content of terephthalate for both the chromium‐ and iron‐containing materials. Furthermore, an S‐shaped adsorption isotherm was visible for all of these mixed‐linker materials. The step steepened and shifted towards lower relative pressures with increasing terephthalate contents. A similar behavior has previously been found for MIL‐53(Al)‐NH_2_(X) materials.^[13]^ However, the position and the steepness of the step in the adsorption isotherms was significantly different for MIL‐53(Al_0.8_M_0.2_)‐NH_2_(X) (M=Cr, Fe) compared to monometallic MIL‐53(Al)‐NH_2_(X) materials.^[13a]^

A clear step in the adsorption isotherm and, thus, a breathing transition was present for the already earlier reported MIL‐53(Al_0.8_V_0.2_)‐NH_2_(100) (Figure 6 b).^[7a]^ Upon the incorporation of terephthalate, the step flattened for MIL‐53(Al_0.8_V_0.2_)‐NH_2_(80) and MIL‐53(Al_0.8_V_0.2_)‐NH_2_(60) and disappeared for MIL‐53(Al_0.8_V_0.2_)‐NH_2_(40). The latter material showed an isotherm, which is typically observed for rigid microporous solids. This observation was in agreement with the powder X‐ray diffraction patterns, which showed that the lp‐form of the MIL‐53 structure dominated with increasing terephthalate content. Thus, the nitrogen physisorption measurements indicated that an inflexible lp‐form seemed to be favorable for MIL‐53(Al_0.8_V_0.2_)‐NH_2_(40). Initially, the scandium analogues were expected to be the least interesting materials during nitrogen physisorption, since MIL‐53(Al_0.8_Sc_0.2_)‐NH_2_(100) did not show any significant uptake of nitrogen in our previous study.^[7a]^ However, the additional incorporation of terephthalate did have a tremendous influence on the nitrogen physisorption isotherms (Figure 6 a). MIL‐53(Al_0.8_Sc_0.2_)‐NH_2_(80) showed only a small adsorbed volume at low relative pressures, but featured a large step in the adsorption isotherm, which we have attributed to a breathing transition resulting in approximately 300 cm^3^g^−1^ of adsorbed nitrogen. Remarkably, the height of this step is larger than the one observed for both MIL‐53(Al)‐NH_2_(100) or MIL‐53(Al_0.8_V_0.2_)‐NH_2_(100). We assume that MIL‐53(Al_0.8_Sc_0.2_)‐NH_2_(80) featured a very narrow pore (vnp) or closed pore (cp) form at low relative pressures and underwent a direct breathing transition to a lp‐form. However, materials with higher amounts of terephthalate in the framework did not show a similar behavior, as no step was observed in the adsorption isotherms of MIL‐53(Al_0.8_Sc_0.2_)‐NH_2_(60) and MIL‐53(Al_0.8_Sc_0.2_)‐NH_2_(40). Nonetheless, a clear difference of the adsorbed volumes in the adsorption and desorption isotherm was found for both materials similar to the already discussed MIL‐53(Al_0.8_Cr_0.2_)‐NH_2_(100) and MIL‐53(Al_0.8_Fe_0.2_)‐NH_2_(100) materials. Thus, we propose that a breathing transition also occurs for MIL‐53(Al_0.8_Sc_0.2_)‐NH_2_(60) and MIL‐53(Al_0.8_Sc_0.2_)‐NH_2_(40) at high relative pressures.

**Figure 7:**
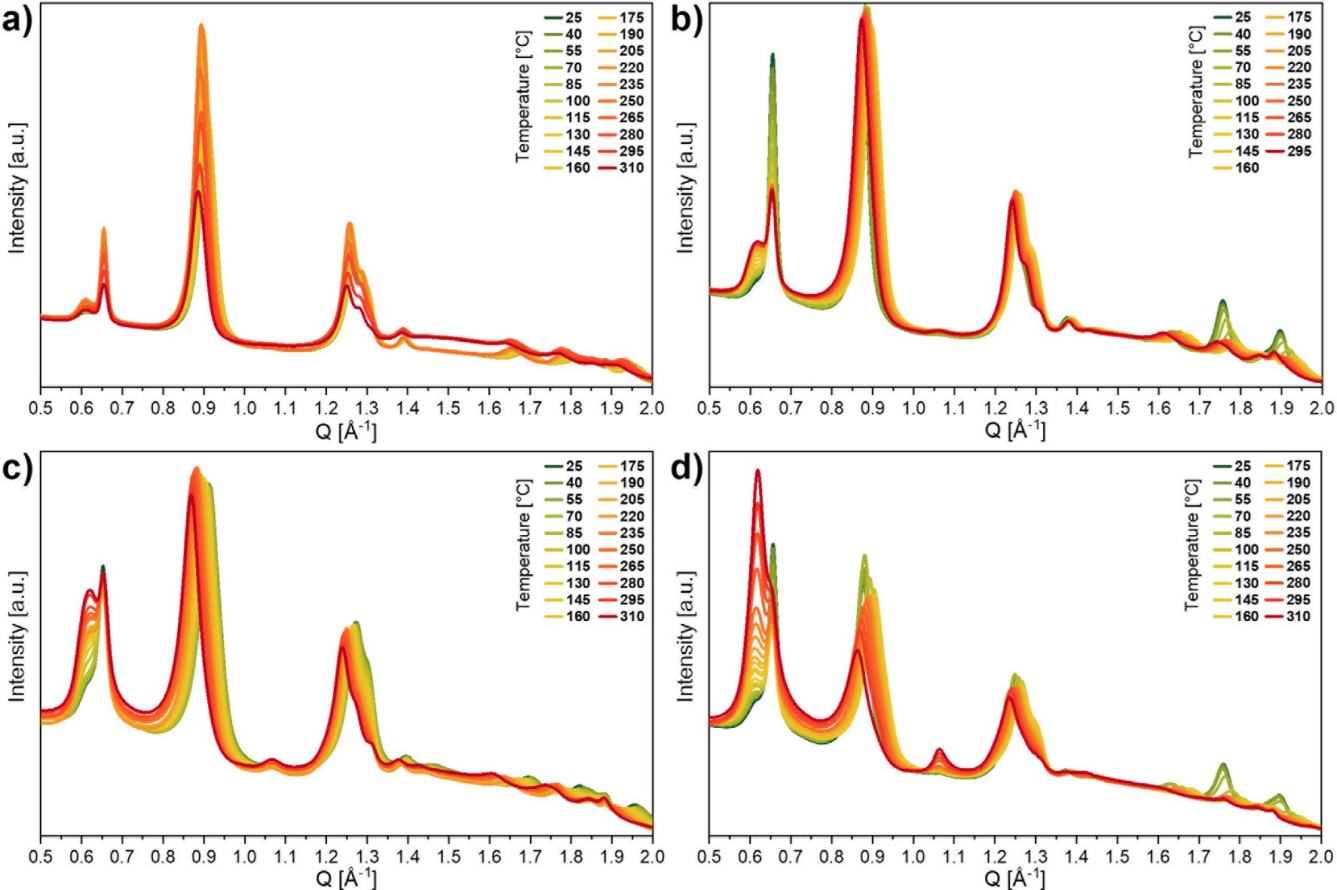
High‐resolution powder X‐ray diffraction patterns of MIL‐53(Al_0.8_Fe_0.2_)‐NH_2_(X) materials recorded in situ at different temperatures under nitrogen at ambient pressure (X=a) 100, b) 80, c) 60 or d) 40).

In situ high‐resolution powder X‐ray diffraction patterns have been recorded for MIL‐53(Al_0.8_Fe_0.2_)‐NH_2_(X) materials in nitrogen flow to investigate their temperature‐dependent breathing behavior. These materials have been inserted as powders into a capillary set‐up and heated to 310 °C using a hot air blower. MIL‐53(Al_0.8_Fe_0.2_)‐NH_2_(100) was heated only to 250 °C, as it started to decompose at higher temperatures. MIL‐53(Al_0.8_Fe_0.2_)‐NH_2_(100) (Figure 7 a) showed only small changes upon heating to 250 °C. With decreasing amount of 2‐aminoterephthalate and increasing amount of terephthalate as linker molecule, the observed changes in the recorded powder diffraction pattern upon heating to 310 °C were more pronounced (Figure 7 b–d). While the position of the (200) reflection at 0.66 Å^−1^ did not change at elevated temperatures, the (110) reflection close to 0.90 Å^−1^ shifted to higher values until 130 °C and subsequently to lower values at higher temperatures. These shifts were more pronounced with increasing terephthalate content. Furthermore, the intensity of the small reflection at 0.61 Å^−1^, which was visible only as a shoulder at room temperature for all MIL‐53(Al_0.8_Fe_0.2_)‐NH_2_(X) materials, increased with increasing temperature and increasing terephthalate content.

Additionally, a reflection at 1.07 Å^−1^ was only observed at elevated temperatures for MIL‐53(Al_0.8_Fe_0.2_)‐NH_2_(60) and MIL‐53(Al_0.8_Fe_0.2_)‐NH_2_(40). The reflections at 0.61 and 1.07 Å^−1^ could be assigned to the (001) and (101) reflections of a lp‐form. The increasing intensity of the (001) reflection indicated that an increasing number of crystallites underwent a np→lp transition at elevated temperatures. Hence, the fraction of the lp‐form increased with increasing terephthalate content. However, no complete phase transition was observed in the available temperature range for any of these materials. Similar tendencies have been observed for MIL‐53(Al_0.8_Cr_0.2_)‐NH_2_(X) materials (Figure S3, lab powder X‐ray diffractometer) and were also reported for monometallic MIL‐53(Al)‐NH_2_(X) materials.^[11]^

**Figure 8:**
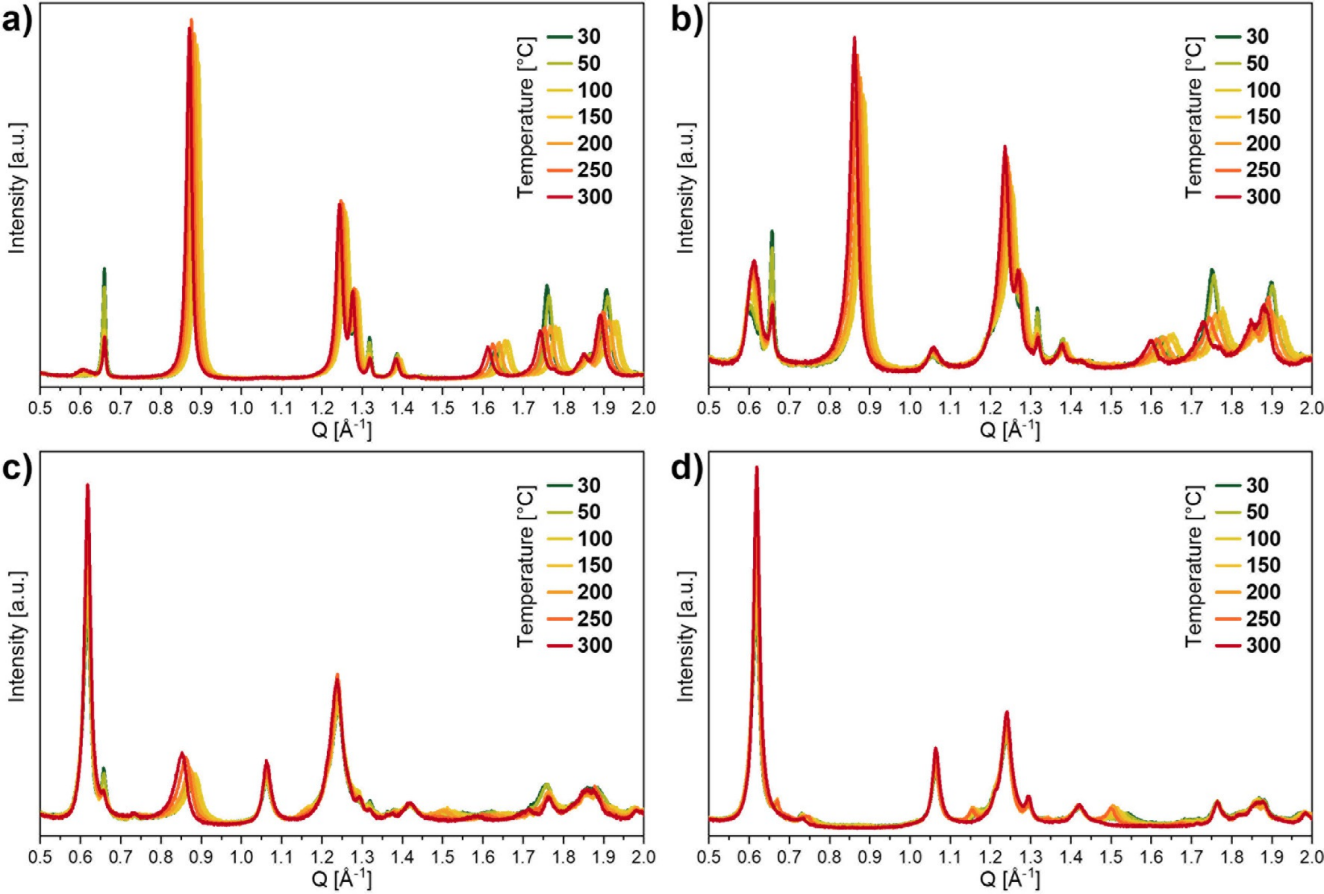
Powder X‐ray diffraction patterns of MIL‐53(Al_0.8_V_0.2_)‐NH_2_(X) materials recorded in situ at different temperatures under nitrogen at ambient pressure with a lab powder X‐ray diffractometer (X=a) 100, b) 80, c) 60 or d) 40).

In the case of the vanadium‐containing MIL‐53(Al_0.8_V_0.2_)‐NH_2_(X) materials, the temperature‐dependent flexibility has been investigated using a lab powder X‐ray diffractometer. The results showed that the diffraction patterns of all materials changed only slightly at elevated temperature in comparison to the patterns at 30 °C (Figure 8). The position of several reflections of the np‐form shifted similarly to the already discussed MIL‐53(Al_0.8_Fe_0.2_)‐NH_2_(X) materials at elevated temperatures (e.g., *Q*=0.88, 1.26, 1.28, 1.63, 1.76 Å^−1^). With increasing amount of terephthalate in the framework, these shifts were slightly more pronounced, which indicated a slightly higher flexibility of the framework structure. The intensity of these reflections did not change significantly, which indicated that the fraction of the np‐phase remained constant over the investigated temperature range. The intensity of the characteristic reflections of the lp‐form (*Q*=0.61, 1.07 Å^−1^) remained also almost constant and their positions did not show any shifts.

Only for MIL‐53(Al_0.8_V_0.2_)‐NH_2_(40), shifts of the small reflections at *Q*=0.76, 1.18, and 1.54 Å^−1^ towards lower values were visible. Furthermore, these reflections were visible up to 250 °C, but not at 300 °C. A possible explanation would be that these reflections originated from a small proportion of a np‐phase, which transformed into a lp‐form above 250 °C, but no unambiguous prove can be provided so far. In summary, presumably no significant np→lp transitions occurred for all MIL‐53(Al_0.8_V_0.2_)‐NH_2_(X) materials at temperatures up to 300 °C, which was particularly surprising for MIL‐53(Al_0.8_V_0.2_)‐NH_2_(60), since already a large fraction of the lp‐phase was present at room temperature and it was expected that the np‐form would easily undergo a transformation into the lp‐from.

**Scheme 2:**
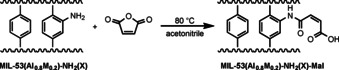
Schematic representation of the post‐synthetic modification of MIL‐53(Al_0.8_M_0.2_)‐NH_2_(X) materials performed with maleic anhydride.

Uncoordinated amine‐groups of metal–organic frameworks are commonly used for post‐synthetic modification reactions.^[14]^ For this purpose, maleic anhydride is a common substrate as the modification proceeds by an addition reaction and no water is eliminated in the course of the modification process.[^13a^, ^14a^, ^14b^, ^15^] This way, chelating side groups or Brønsted acid functionalities can be introduced within the pores. The chelating group is particularly interesting for the immobilization of metal ions to obtain single‐site catalyst materials.[^3a^, ^13a^, ^15b^, ^16^] Therefore, all MIL‐53(Al_0.8_M_0.2_)‐NH_2_(X) materials were modified with maleic anhydride (Scheme 2) to show that the mixed‐metal/mixed‐linker concept is compatible with post‐synthetic modifications and to investigate whether different combinations or ratios of components have an influence on the modification process. In analogy to our previous study,^[7a]^ all materials were activated at 130 °C in vacuum before adding a solution of maleic anhydride in acetonitrile to the dehydrated materials.

The metal ratios of all materials did not change during the post‐synthetic modification process (Table S2). The number of functionalized amine linkers (modification degree) was determined based on the recorded ^1^H NMR spectra of digested materials. The recorded spectra (example shown in Figure S4) showed signals originating from protons of both terephthalate (*δ*=7.85 ppm (s, 4 H, H_4_)) and unmodified 2‐aminoterephthalate (*δ*=7.16 (d, 1 H, H_1b_), 7.24 (s, 1 H, H_1c_), 7.67 ppm (d, 1 H, H_1a_)) linkers. Furthermore, three signals of protons from the benzene ring of modified 2‐aminoterephthalate linkers were visible (*δ*=7.59 (d, 1 H, H_2b_), 7.85 ppm (d, 1 H, H_2a_), 8.58 (s, 1 H, H_2c_)), of which the signal with the highest downfield shift overlapped with the strong singlet of terephthalate. The protons of the maleate side group of the modified linker were visible as two individual signals (*δ*=6.08 (d, 1 H, H_2e_), 6.49 ppm (d, 1 H, H_2d_)). In addition, a singlet at *δ*=5.98 ppm (s, 2 H, H_3_) was ascribed to free maleate molecules. Because a strong acid (DCl) and a strong base (NaOD) were involved in the digestion process, these free maleate molecules were most likely formed during the digestion process by cleavage of the amide band of the post‐synthetically modified linker. A measurement of the prepared solution after four weeks also supported this assumption, since the number of free maleate molecules relative to the number of modified linkers increased (Figure S6), thus, indicating an ongoing cleavage of the amide bond. As the recorded ATR‐IR spectra after post‐synthetic modification did not show any bands of free maleic acid or maleic anhydride within the pores (characteristic bands expected close to 2900 and 1780 cm^−1^, respectively), it is assumed that all free maleate molecules found in the NMR spectra originated from the cleavage of post‐synthetically modified linker molecules.

The determined percentages of modified amine groups (Table S5) were in the range 16–86 %. Only for the scandium‐containing materials, a clear correlation of the modification degree and the linker ratio was found. The number of modified amine groups increased with an increasing fraction of terephthalate in the framework. The overall modification degree of mixed‐metal/mixed‐linker materials, which is the number of modified linker molecules with respect to the total number of linkers (terephthalate + 2‐aminoterephthalate), was in the range 16–36 %.

**Figure 9:**
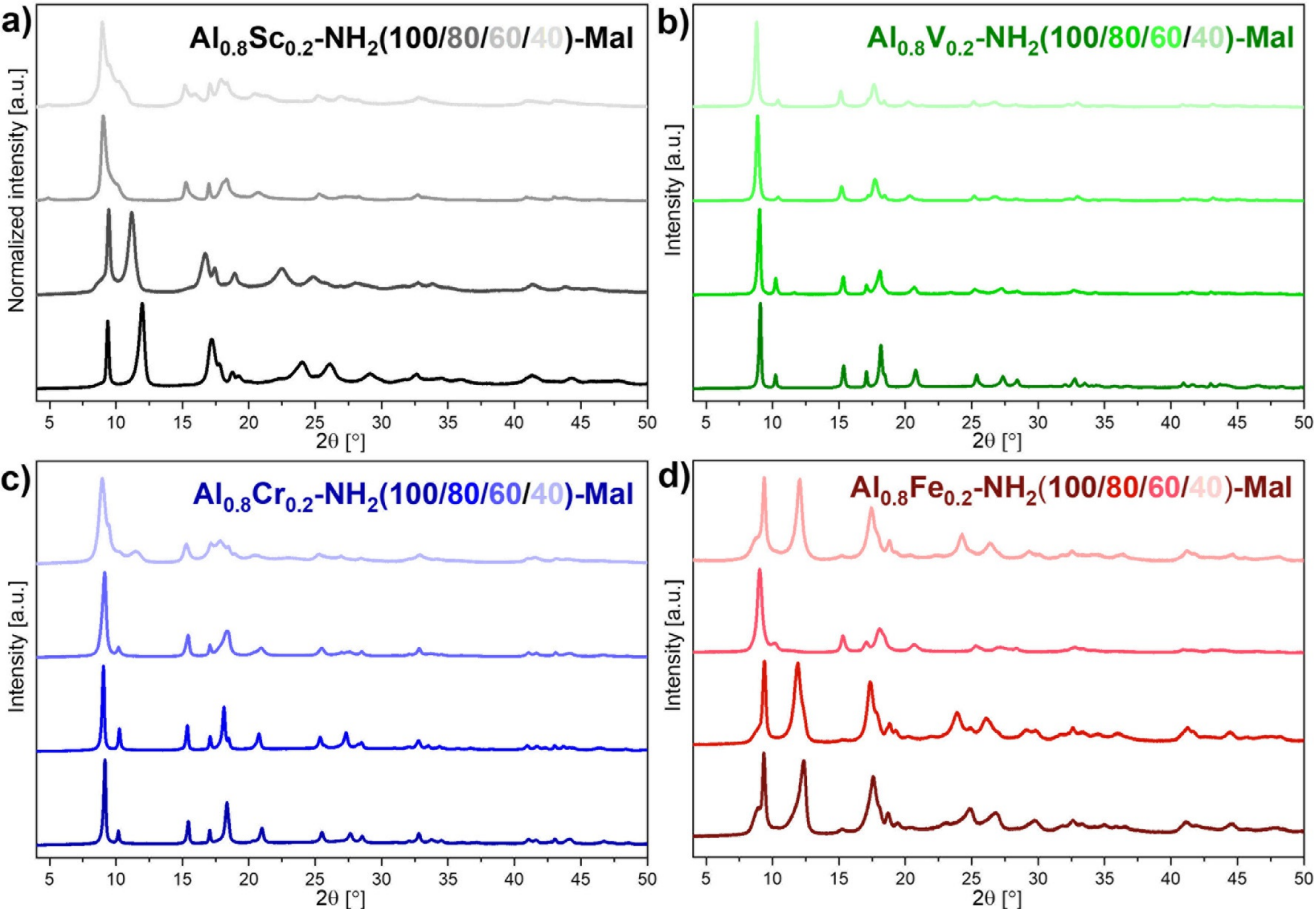
Powder X‐ray diffraction patterns of post‐synthetically modified MIL‐53(Al_0.8_M_0.2_)‐NH_2_(X)‐Mal materials (X=100, 80, 60, 40).

The recorded powder X‐ray diffraction patterns (Figure 9) confirmed that the MIL‐53 structure remained intact during the post‐synthetic modification process for all materials. The characteristic reflections of either np‐ or lp‐forms were clearly visible. In contrast to the diffraction patterns before the modification (Figure 3), for which only few materials showed reflections of lp‐phases, most materials were found to be in a lp‐form after post‐synthetic modification (one characteristic intense reflection close to 2*θ*=8.7°). However, the iron‐containing materials were found predominantly in the np‐form (two characteristic reflections close to 2*θ*=9.4 and 12.2°). The transition of the np‐form to the lp‐form during the post‐synthetic modification process seemed to be a result of the space requirement of the incorporated maleate side group.

The recorded diffraction patterns were used to determine the unit cell parameters after the modification process by using Pawley refinements. The obtained pore dimensions confirmed the qualitatively derived assumption on the presence of np‐ and lp‐forms (Table S4). However, conclusive refinements were not possible for all materials, since asymmetric and/or broadened reflections were present for several materials, which presumably resulted from non‐uniform modification within the crystallites.

**Figure 10:**
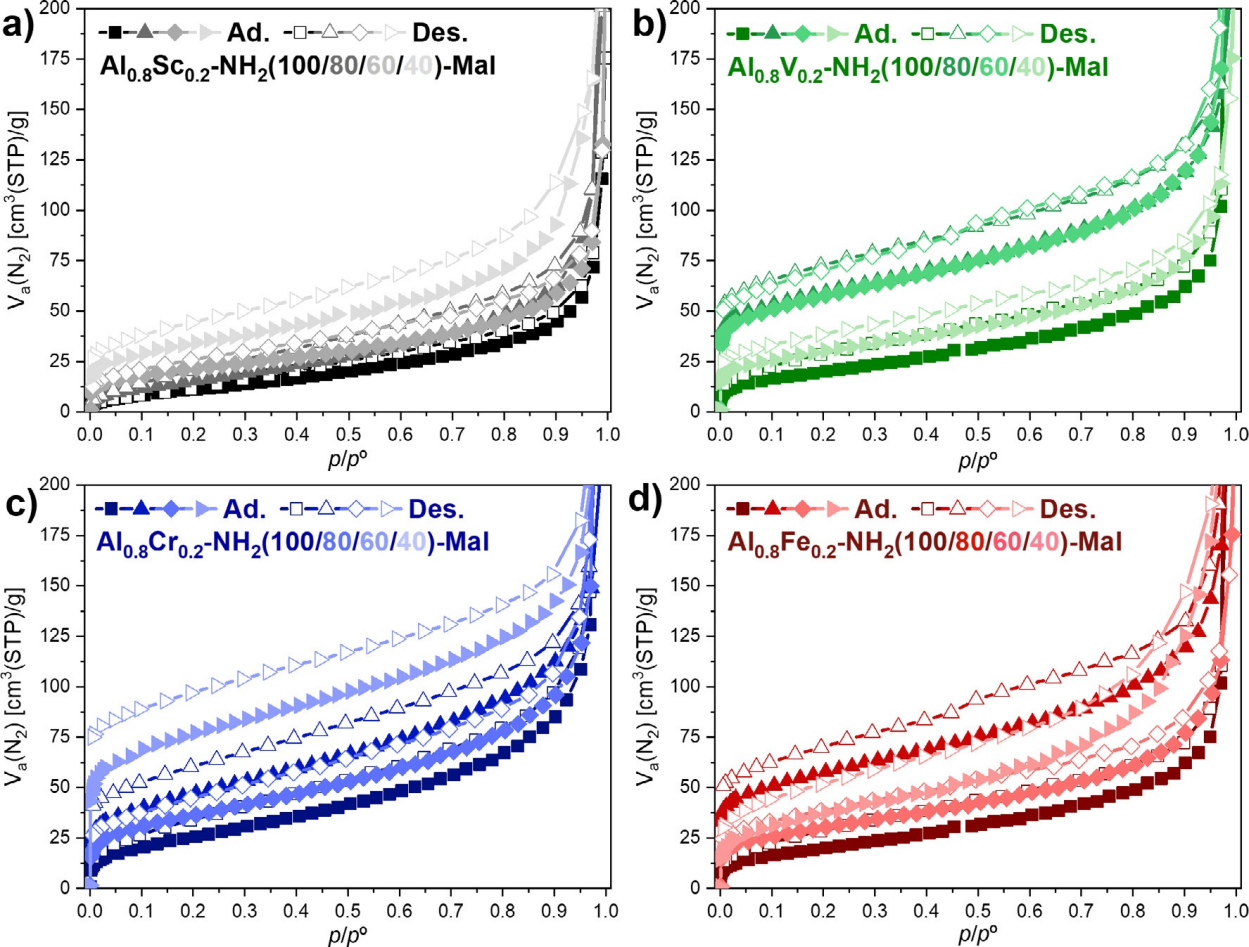
Nitrogen physisorption isotherms of post‐synthetically modified MIL‐53(Al_0.8_M_0.2_)‐NH_2_(X)‐Mal materials (X=100, 80, 60, 40) measured at 77 K.

Nitrogen physisorption isotherms after post‐synthetic modification showed dramatically decreased nitrogen uptakes in comparison to the pristine MIL‐53(Al_0.8_M_0.2_)‐NH_2_(X) materials (cf. Figures 6 and 10). Furthermore, no S‐shaped adsorption isotherms were found, which indicated that none of these materials showed any flexibility under the applied measurement conditions anymore. The reduced nitrogen uptake can be explained by the incorporation of the maleate side groups, which require space in the pores and, thus, reduce the accessible pore volume. A small gap between the adsorption and desorption branches reaching to the lowest measured relative pressure was visible for several materials, but an unambiguous explanation cannot be provided so far. Whereas the specific surface areas (BET method) were in the range of 30–260 m^2^ g^−1^, the micropore volumes (t‐plot method) were relatively small (≤0.05 cm^3^ g^−1^, Table S5); this suggested that the majority of the accessible surface area resulted from interparticle void space.

## Conclusions

This study has shown that mixed‐metal/mixed‐linker MIL‐53(Al_0.8_M_0.2_)‐NH_2_(X) materials were successfully synthesized by using a direct synthesis procedure at ambient pressure. The thorough characterization revealed that all components were statistically distributed within the MIL‐53 framework structure with ratios close to the expected values, which were applied during the synthesis. Based on the powder X‐ray diffraction patterns, the framework structures continuously changed with varying linker ratios. Whereas the np‐form was dominant for the majority of the materials, reflections of a lp‐form appeared and increased in intensity with higher terephthalate content for the MIL‐53(Al_0.8_Cr_0.2_)‐NH_2_(X) materials. Furthermore, the lp‐form was the dominant phase for MIL‐53(Al_0.8_V_0.2_)‐NH_2_(60) and exclusively present for MIL‐53(Al_0.8_V_0.2_)‐NH_2_(40). The thermal stability strongly depended on the linker ratio for all metal combinations and increased with increasing terephthalate content. Nitrogen physisorption measurements showed that the breathing behavior strongly depended on the linker ratio of terephthalate and 2‐aminoterephthalate. An increased adsorption volume with increasing terephthalate content was observed for all metal combinations. Remarkably, most of the adsorption isotherms of mixed‐metal/mixed‐linker materials showed a step that was characteristic for the breathing behavior and was not observed for most single‐linker MIL‐53(Al_0.8_M_0.2_)‐NH_2_(100) materials containing only 2‐aminoterephthalate. The height, the position and the steepness of this step depended on both the metal combination and the linker ratio. Temperature‐dependent powder X‐ray diffraction measurements showed that only small changes occurred in the framework structure between room temperature and 300 °C and that no complete np→lp transition could be achieved. All developed materials were used for post‐synthetic modification reactions with maleic anhydride. The framework structure remained intact during the modification process, but most materials adopted a lp‐form after the modification. Due to the presence of the maleate side group, the nitrogen uptake decreased significantly after the modification. Thus, the properties of the MIL‐53 structure can be carefully tuned by selecting appropriate metal combinations and linker ratios.

## Experimental Section

All chemicals were purchased and used as received without further purification. Demineralized water was used for all procedures. Chemicals were used with the following purities: *N*,*N*‐dimethylformamide (99.9 %), 2‐aminoterephthalic acid (99 %), terephthalic acid (98 %), aluminum(III) nitrate nonahydrate (≥98 %), iron(III) chloride hexahydrate (≥98 %), scandium(III) chloride (99.99 % Sc), chromium(III) nitrate nonahydrate (≥98 %), vanadium(III) chloride (≥97 %), concentrated hydrochloric acid (HCl, 37 wt %), maleic anhydride (≥99 %), acetonitrile (≥99.5 %). The denomination of all materials refers to the ratios of the components that were used for the synthesis.

**Synthesis of MIL‐53(Al_0.8_Sc_0.2_)‐NH_2_(X) materials**: 2‐Aminoterephthalic acid and terephthalic acid were dissolved in 10 mL of water and 10 mL of *N*,*N*‐dimethylformamide (DMF) at 90 °C with stirring. After complete dissolution, a solution containing aluminum(III) nitrate nonahydrate (0.7998 g, 2.13 mmol, 0.80 equiv.) and scandium(III) chloride (0.0806 g, 0.53 mmol, 0.20 equiv.) in 5 mL of water was added under continuous stirring. The resulting reaction solution was stirred at 90 °C under reflux cooling for 72 h. Afterwards, the hot suspension was filtrated by using a glass filter, washed (3×20 mL DMF, 1×30 mL H_2_O) and dried in air (at room temperature overnight and at 130 °C for another 3 days). Table 1 contains the used amounts of 2‐aminoterephthalic acid and terephthalic acid.

**Table 1:** Masses and molar amounts of linkers that were used for the synthesis of MIL‐53(Al_0.8_M_0.2_)‐NH_2_(X) materials. M=Sc, V, Cr or Fe and X=100, 80, 60 or 40.

	2‐Aminoterephthalic acid	Terephthalic acid
Al_0.8_M_0.2_‐NH_2_(100)	0.4828 g 2.67 mmol 1.00 equiv.	–
Al_0.8_M_0.2_‐NH_2_(80)	0.3862 g 2.13 mmol 0.80 equiv.	0.0885 g 0.53 mmol 0.20 equiv.
Al_0.8_M_0.2_‐NH_2_(60)	0.2897 g 1.60 mmol 0.60 equiv.	0.1771 g 1.07 mmol 0.40 equiv.
Al_0.8_M_0.2_‐NH_2_(40)	0.1931 g 1.07 mmol 0.40 equiv.	0.2656 g 1.60 mmol 0.60 equiv.

**Synthesis of MIL‐53(Al_0.8_V_0.2_)‐NH_2_(X) materials**: 2‐Aminoterephthalic acid and terephthalic acid were dissolved in 10 mL of water and 10 mL of *N*,*N*‐dimethylformamide (DMF) at 90 °C with stirring. After complete dissolution, a solution containing aluminum(III) nitrate nonahydrate (0.7998 g, 2.13 mmol, 0.80 equiv.) and vanadium(III) chloride (0.0838 g, 0.53 mmol, 0.20 equiv.) in 5 mL of water was added under continuous stirring. The resulting reaction solution was stirred at 90 °C under reflux cooling for 72 hours. Afterwards, the hot suspension was filtrated by using a glass filter, washed (3×20 mL DMF, 1×30 mL H_2_O) and dried in air atmosphere (overnight at room temperature and for another 3 days at 130 °C). Table 1 contains the used amounts of 2‐aminoterephthalic acid and terephthalic acid. To remove residual linker molecules from within the pores, the obtained materials were suspended in 20 mL of DMF at 90 °C for 4 h, subsequently filtrated by using a glass filter, washed (2×20 mL DMF, 1×20 mL H_2_O) and dried in air (at room temperature overnight and at 130 °C for another 3 days).

**Synthesis of MIL‐53(Al_0.8_Cr_0.2_)‐NH_2_(X) materials**: 2‐Aminoterephthalic acid and terephthalic acid were dissolved in 10 mL of water and 10 mL of *N*,*N*‐dimethylformamide (DMF) at 90 °C with stirring. After complete dissolution, a solution containing aluminum(III) nitrate nonahydrate (0.7998 g, 2.13 mmol, 0.80 equiv.), chromium(III) nitrate nonahydrate (0.2133 g, 0.53 mmol, 0.20 equiv.) and concentrated HCl (2.67 mmol, 0.22 mL, 1.00 equiv.) in 5 mL of water was added under continuous stirring. The resulting reaction solution was stirred at 90 °C under reflux cooling for 72 h. Afterwards, the hot suspension was filtrated by using a glass filter, washed (3×20 mL DMF, 1×30 mL H_2_O) and dried in air (at room temperature overnight and at 130 °C for another 3 days). Table 1 contains the used amounts of 2‐aminoterephthalic acid and terephthalic acid.

**Synthesis of MIL‐53(Al_0.8_Fe_0.2_)‐NH_2_(X) materials**: 2‐Aminoterephthalic acid and terephthalic acid were dissolved in 20 mL of water and 20 mL of *N*,*N*‐dimethylformamide (DMF) at 90 °C with stirring. After complete dissolution, a solution containing aluminum(III) nitrate nonahydrate (0.7998 g, 2.13 mmol, 0.80 equiv.) and iron(III) chloride hexahydrate (0.1443 g, 0.53 mmol, 0.20 equiv.) in 10 mL of water was added under continuous stirring. The resulting reaction solution was stirred at 90 °C under reflux cooling for 120 h. Afterwards, the hot suspension was filtrated by using a glass filter, washed (3×20 mL DMF, 1×30 mL H_2_O) and dried in air (at room temperature overnight and at 130 °C for another 3 days). Table 1 contains the used amounts of 2‐aminoterephthalic acid and terephthalic acid.

**Post‐synthetic modification**: MIL‐53(Al_0.8_M_0.2_)‐NH_2_(X) (0.1000 g, ≈0.40 mmol, 1.00 equiv.; M=Sc, V, Cr, Fe; X=100, 80, 60, 40) was heated to 130 °C under vacuum for 4 h to remove water from the pores. The dehydrated MIL‐53(Al_0.8_M_0.2_)‐NH_2_(X) material was cooled to room temperature and, subsequently, a solution of maleic anhydride (0.1569 g, 1.60 mmol, 4.00 equiv.) in 10 mL of acetonitrile was added to the powder under argon atmosphere. This suspension was placed in a self‐made shaker at 80 °C for 24 h. The resulting hot suspension was filtrated by using a glass filter, washed (3×10 mL acetonitrile) and dried in air at room temperature.

**Powder X‐ray diffraction**: Powder X‐ray diffraction patterns were collected using a Bruker D8 Discover powder diffractometer with Cu_Kα_ radiation in the 2*θ* range from 4 to 50° with a step width of 2*θ*=0.0102° and an accumulation time of 1.60 s step^−1^.

The assignment of lattice planes to reflections in the measured powder X‐ray diffraction patterns was performed based on available single crystal data of MIL‐53(Al) (np: CCDC 220477, lp: CCDC 220476).^[17]^

**In situ powder X‐ray diffraction**: Temperature‐dependent in situ powder X‐ray diffraction measurements of MIL‐53(Al_0.8_Cr_0.2_)‐NH_2_(X) and MIL‐53(Al_0.8_V_0.2_)‐NH_2_(X) materials were performed on the Bruker D8 Advance powder diffractometer recorded with Cu_Kα_ radiation in Bragg–Brentano geometry. An in situ measurement chamber XRK 900 from Anton Paar was used with a continuous gas flow of nitrogen. The samples were heated from 30 to 300 °C in intervals of 50 K (30, 50, 100, … 300 °C). Fast diffraction patterns (range: 2*θ*=4 to 50 °, step width: 2*θ*=0.0204°, time/step=0.100 s) were recorded after an equilibration time (25 min) at each temperature step.

Temperature‐dependent high‐resolution in situ powder X‐ray diffraction measurements of MIL‐53(Al_0.8_Fe_0.2_)‐NH_2_(X) materials were performed at PETRA III Extension beamline P24 (operated at 20 keV=0.62 Å) at DESY (Deutsches Elektronensynchrotron in Hamburg, Germany). The samples were inserted in an in situ capillary setup, which was attached to a Kappa‐diffractometer. A continuous stream of nitrogen (100 mL min^−1^) was passed through the capillary. For temperature‐dependent measurements, the samples in the capillaries were heated in steps of 15 K from room temperature to a maximum of 310 °C with a hot air blower. Diffraction patterns were collected for each temperature step after an equilibration time of 5 min.

For a better comparability of the measurements at the lab diffractometer and at the synchrotron, all in situ recorded diffraction patterns are presented in reciprocal space (*Q*).

**Structure refinements**: The structure refinement was performed using TOPAS 4.2. The complete measured range 2*θ*=4.0 to 50.0° of the PXRD measurements was used for the refinement. The Pawley method was used to determine the unit cell parameters starting from reported values of MIL‐53(Al)‐NH_2_(*lt*) (space group *Cc*),^[10]^ MIL‐47(V) (space group *Pnma*)^[12]^ or MIL‐53(Cr)*ht* (space group *Imma*).^[18]^

**Infrared spectroscopy**: ATR‐IR spectra were recorded on a Nicolet 6700 FTIR spectrometer from ThermoFisher Scientific equipped with a Smart iTX ATR‐IR accessory with Ge crystal and a MCT detector operating at liquid nitrogen temperature. A scan range of 400–4000 cm^−1^ with a resolution of 4 cm^−1^ was chosen and 400 spectra were accumulated for each measurement.

**NMR spectroscopy**: Liquid‐phase ^1^H NMR spectra were recorded on a Bruker Ascend 400 MHz spectrometer. Chemical shifts were referenced to internal solvent resonances and reported relative to tetramethylsilane (TMS). However, due to slightly different pH values of the prepared solutions, the residual solvent signal shifted slightly, which led to minor shifts of peaks. Scandium‐, vanadium‐ and chromium‐containing materials were digested prior to the measurement using the following procedure: 5 mg of the MOF material were digested by adding 40 μL of DCl (20 wt % in D_2_O) followed by 5 min of ultrasonication and, subsequently, the addition of 70 μL of NaOD (30 wt % in D_2_O) and 590 μL of D_2_O.

For the iron‐containing materials, a slightly modified procedure was necessary: 50 μL of DCl (20 wt % in D_2_O) were added to 5 mg of MOF material and the mixture was treated for several minutes in an ultrasound bath. Afterwards, 140 μL of NaOD (30 wt % in D_2_O) and 140 μL of D_2_O were added and the mixture was placed in the ultrasound bath for several minutes. The suspension was centrifuged at 10,000 min^−1^ for 2 min and the clear liquid was filled in an NMR tube. 50 μL NaOD (30 wt % in D_2_O) and 150 μL of D_2_O were added to the residual solid, the mixture was placed in the ultrasound bath, centrifuged and the clear liquid was combined with the previously separated liquid in the NMR tube.

The degree of post‐synthetically modified amine groups was calculated based on the following Equation 1 after a careful evaluation of other characterization data as outlined in the Results and Discussions. The assignments of the protons are visualized in Figure S4.

(1) $$\mathrm{Degree}\;\mathrm{of}\;\mathrm{modification}=\frac{H_{2d}+\frac{H_{3}}{2}}{H_{2d}+\frac{H_{1c}+H_{1b}}{2}}$$

For the calculation of the overall modification degree, the calculated degree of modification obtained from Equation 1) was multiplied by the percentage of 2‐aminoterephthalate with respect to the total amount of linkers.

**Thermogravimetric analysis**: The thermal stability and the decomposition of the materials was investigated in oxidative atmosphere (20 % O_2_/He) on a thermo balance Cahn TG‐2131. 15 mg of sample were heated from 40 to 1000 °C with a heating ramp of 5 K min^−1^.

**Nitrogen physisorption**: The samples were activated for 20 h at 130 °C prior to the nitrogen physisorption measurements. The measurements were performed on an Autosorb 6 setup from Quantachrome. Micropore volumes were estimated by using the t‐plot method^[19]^ and specific surface areas by using the BET method.^[20]^

**Elemental analysis**: Metal ratios were determined using ICP‐OES (inductively coupled plasma optical emission spectroscopy). An iCAP 6500 Duo from Thermo Scientific with standard equipment was used for the measurements. A six‐point standard was used for the calibration curve. Prior to the measurement, the samples were dissolved in diluted nitric acid. The data analysis was performed with the device's own software “iTEVA9.8”.

**X‐ray absorption spectroscopy**: XAS experiments were performed at PETRA III Extension beamline P65 (energy range: 4–44 keV) at DESY.^[21]^ For the measurements at the Fe K‐edge, a Si(111) C‐type double crystal monochromator was used. The beam current was 100 mA with a ring energy of 6.08 GeV. All samples were prepared as pellets using cellulose as a binder. All spectra were recorded in continuous scan mode both in transmission and in fluorescence mode at ambient temperature and pressure in the range of −150 to 1000 eV around the edge within 180 s. For the data analysis, transmission data were used. For calibration, an Fe foil was measured as a reference simultaneously with the samples.

The data treatment was performed using the Demeter software package.^[22]^ In order to compensate for the oversampling of the continuous scan mode, the data points of the obtained spectra were reduced with the help of the “rebin” function of the Athena software (edge region: −50 to +50 eV; pre‐edge grid: 5 eV; XANES grid: 0.5 eV; EXAFS grid: 0.05 Å^−1^). For data evaluation, a Victoreen‐type polynomial was subtracted from the spectrum to remove the background using the Athena software. The first inflection point of the edge was taken as edge energy *E*_0_.

## Conflict of interest

The authors declare no conflict of interests.

## Supporting information

As a service to our authors and readers, this journal provides supporting information supplied by the authors. Such materials are peer reviewed and may be re‐organized for online delivery, but are not copy‐edited or typeset. Technical support issues arising from supporting information (other than missing files) should be addressed to the authors.

Supplementary
