# Supplementary material for: Increasing the Complexity in the MIL‐53 Structure: The Combination of the Mixed‐Metal and the Mixed‐Linker Concepts
Source: Chemistry. 2020 Dec 14;27(5):1724–35. doi: 10.1002/chem.202003304 (PMC7898851; doi:10.1002/chem.202003304)
Supplement: Supplementary file 1 — Supplementary [file CHEM-27-1724-s001.pdf]

Chemistry–A European Journal

Supporting Information

Increasing the Complexity in the MIL-53 Structure: The Combination of the Mixed-Metal and the Mixed-Linker Concepts

Johannes Bitzer, Milada Teubnerová, and Wolfgang Kleist*[a]

1. Determined linker ratios

Table S1. Comparison of expected and experimentally determined linker ratios using liquid-phase ^{1}H-NMR spectroscopy of digested materials. The corresponding spectra are shown in **Figure S1**.

	2-aminoterephthalate/terephthalate ratio	
	**expected**	**determined**
$Al_{0.8}Sc_{0.2}$-NH_2(100)	100 : 0	-
$Al_{0.8}Sc_{0.2}$-NH_2(80)	80 : 20	79 : 21
$Al_{0.8}Sc_{0.2}$-NH_2(60)	60 : 40	60 : 40
$Al_{0.8}Sc_{0.2}$-NH_2(40)	40 : 60	40 : 60
$Al_{0.8}V_{0.2}$-NH_2(100)	100 : 0	-
$Al_{0.8}V_{0.2}$-NH_2(80)	80 : 20	78 : 22
$Al_{0.8}V0_{.2}$-NH_2(60)	60 : 40	58 : 42
$Al_{0.8}V_{0.2}$-NH_2(40)	40 : 60	37 : 63
$Al_{0.8}Cr_{0.2}$-NH_2(100)	100 : 0	-
$Al_{0.8}Cr_{0.2}$-NH_2(80)	80 : 20	79 : 21
$Al_{0.8}Cr_{0.2}$-NH_2(60)	60 : 40	59 : 41
$Al_{0.8}Cr_{0.2}$-NH_2(40)	40 : 60	40 : 60
$Al_{0.8}Fe_{0.2}$-NH_2(100)	100 : 0	-
$Al_{0.8}Fe_{0.2}$-NH_2(80)	80 : 20	78 : 22
$Al_{0.8}Fe_{0.2}$-NH_2(60)	60 : 40	60 : 40
$Al_{0.8}Fe_{0.2}$-NH_2(40)	40 : 60	42 : 58

2. ^{1}H-NMR spectra of MIL-53($Al_{0.8}M_{0.2}$)-NH_2(X) materials

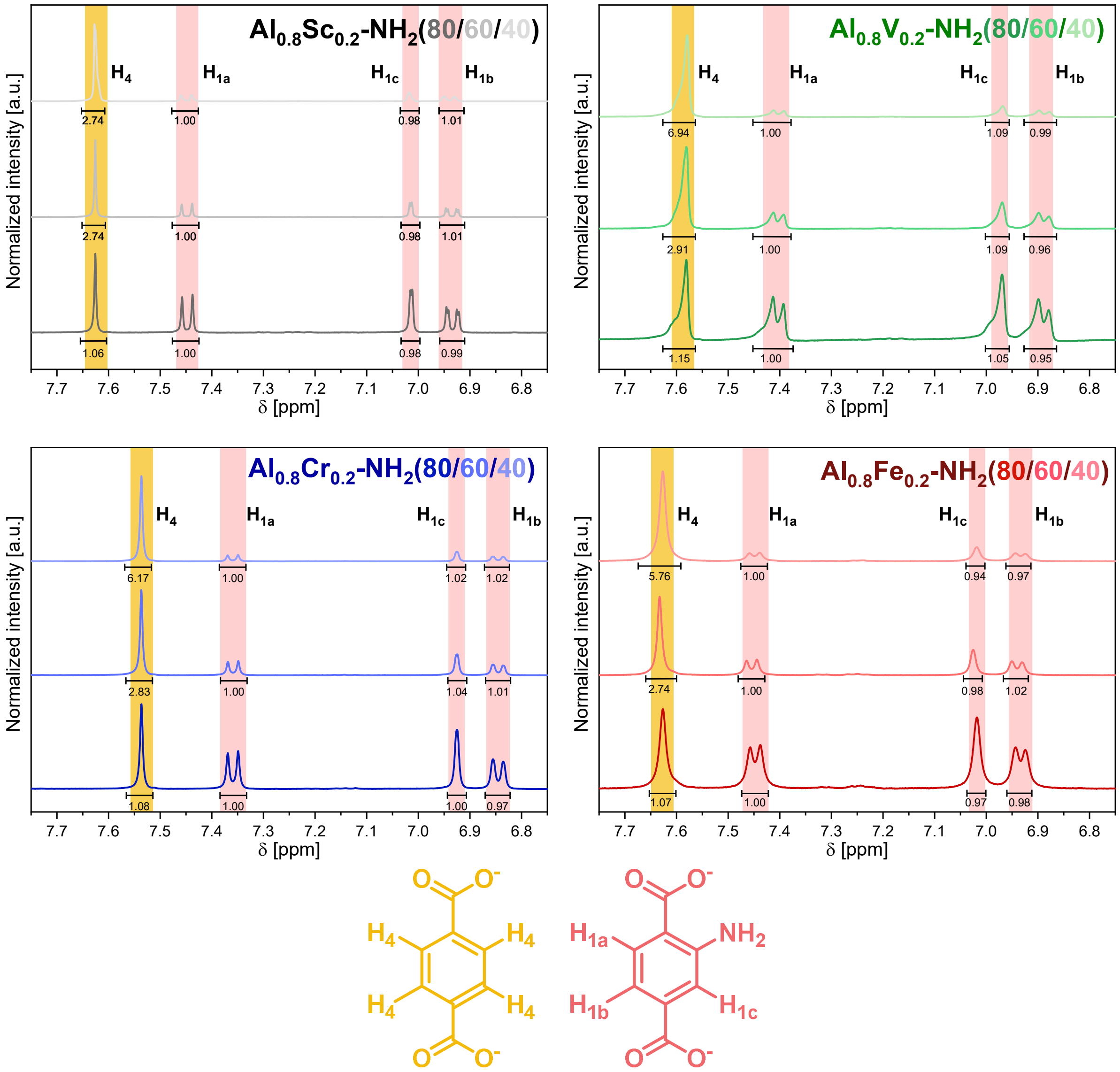

Figure S1. ^{1}H-NMR spectra of digested MIL-53($Al_{0.8}M_{0.2}$)-NH_2(X) materials and the corresponding assignment of protons.

3. Determined metal ratios

Table S2. Comparison of the metal ratios, which were present during synthesis (expected), and the determined values from ICP-OES measurements of digested materials after their synthesis (MIL-53($Al_{0.8}M_{0.2}$)-NH_2(X)) and after the post-synthetic modification (MIL-53($Al_{0.8}M_{0.2}$)-NH_2(X)-Mal).

	**Al : M ratio**		
	**expected**	**determined**	
		**after synthesis**	**after PSM**
$Al_{0.8}Sc_{0.2}$-NH_2(100)	0.80 : 0.20	0.68 : 0.32	0.69 : 0.31
$Al_{0.8}Sc_{0.2}$-NH_2(80)	0.80 : 0.20	0.69 : 0.31	0.70 : 0.30
$Al_{0.8}Sc_{0.2}$-NH_2(60)	0.80 : 0.20	0.68 : 0.32	0.70 : 0.30
$Al_{0.8}Sc_{0.2}$-NH_2(40)	0.80 : 0.20	0.68 : 0.32	0.69 : 0.31
$Al_{0.8}V_{0.2}$-NH_2(100)	0.80 : 0.20	0.86 : 0.14	0.85 : 0.15
$Al_{0.8}V_{0.2}$-NH_2(80)	0.80 : 0.20	0.84 : 0.16	0.85 : 0.15
$Al_{0.8}V_{0.2}$-NH_2(60)	0.80 : 0.20	0.82 : 0.18	0.84 : 0.16
$Al_{0.8}V_{0.2}$-NH_2(40)	0.80 : 0.20	0.83 : 0.18	0.84 : 0.16
$Al_{0.8}Cr_{0.2}$-NH_2(100)	0.80 : 0.20	0.82 : 0.18	0.83 : 0.17
$Al_{0.8}Cr_{0.2}$-NH_2(80)	0.80 : 0.20	0.82 : 0.18	0.83 : 0.17
$Al_{0.8}Cr_{0.2}$-NH_2(60)	0.80 : 0.20	0.82 : 0.18	0.83 : 0.17
$Al_{0.8}Cr_{0.2}$-NH_2(40)	0.80 : 0.20	0.83 : 0.17	0.85 : 0.15
$Al_{0.8}Fe_{0.2}$-NH_2(100)	0.80 : 0.20	0.81 : 0.19	0.83 : 0.17
$Al_{0.8}Fe_{0.2}$-NH_2(80)	0.80 : 0.20	0.81 : 0.19	0.82 : 0.18
$Al_{0.8}Fe_{0.2}$-NH_2(60)	0.80 : 0.20	0.81 : 0.19	0.82 : 0.18
$Al_{0.8}Fe_{0.2}$-NH_2(40)	0.80 : 0.20	0.81 : 0.19	0.83 : 0.17

4. PXRD patterns and ATR-IR spectra of *as*-synthesized MIL-53($Al_{0.8}V_{0.2}$)-NH_2(X) materials

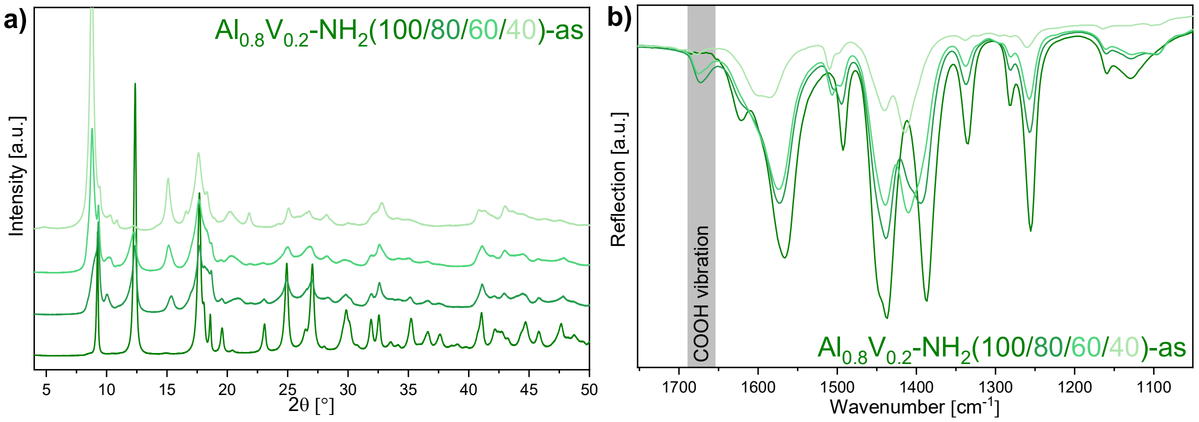

Figure S2. Powder X-ray diffraction patterns (**a**) and ATR-IR spectra (**b**) of *as*-synthesized MIL-53($Al_{0.8}V_{0.2}$)-NH_2(X) materials before performing the washing procedure.

5. Refinement results of MIL-53($Al_{0.8}M_{0.2}$)-NH_2(X) materials

Table S3. Results of the performed unit cell refinements (Pawley method) of MIL-53($Al_{0.8}M_{0.2}$)-NH_2(X) materials. For M = Sc and Fe, only a *np*-structure similar to MIL-53(Al)-NH_2 was considered, while a *lp*-structure was used for V- and Cr-containing materials in addition to the *np*-structure.

	space group	a [Å]	b [Å]	c [Å]	β [°]	R_{wp} [%]
$Al_{0.8}Sc_{0.2}$-NH_2(100)	*Cc*	19.934(9)	7.724(2)	6.725(1)	105.97(3)	6.33
$Al_{0.8}Sc_{0.2}$-NH_2(80)	*Cc*	19.886(8)	7.696(2)	6.714(1)	105.74(3)	8.00
$Al_{0.8}Sc_{0.2}$-NH_2(60)	*Cc*	19.827(6)	7.701(1)	6.687(1)	105.48(2)	11.53
$Al_{0.8}Sc_{0.2}$-NH_2(40)	*Cc*	10.799(9)	7.704(2)	6.750(2)	105.31(3)	7.04
$Al_{0.8}V_{0.2}$-NH_2(100)	*Cc*	19.787(04)	7.720(01)	6.607(01)	105.48(01)	5.74
$Al_{0.8}V_{0.2}$-NH_2(80)	*Cc*	19.636(13)	7.718(03)	6.581(02)	105.52(08)	7.60*
	Pnma	6.643(04)	17.145(14)	12.711(10)	90.00	
$Al_{0.8}V_{0.2}$-NH_2(60)	*Cc*	19.678(75)	7.780(04)	6.602(14)	106.09(81)	8.14*
	Pnma	6.656(02)	17.081(11)	12.489(13)	90.00	
$Al_{0.8}V_{0.2}$-NH_2(40)	*Pnma*	6.648(02)	17.002(07)	12.500(04)	90.00	10.33
$Al_{0.8}Cr_{0.2}$-NH_2(100)	*Cc*	19.778(06)	7.738(01)	6.602(01)	105.56(02)	4.50
$Al_{0.8}Cr_{0.2}$-NH_2(80)	*Cc*	19.649(08)	7.723(02)	6.576(02)	105.20(05)	7.13*
	Imma	6.602(14)	17.307(24)	12.669(16)	90.00	
$Al_{0.8}Cr_{0.2}$-NH_2(60)	*Cc*	19.638(12)	7.722(03)	6.579(03)	105.14(07)	7.20*
	Imma	6.612(06)	17.270(19)	12.586(11)	90.00	
$Al_{0.8}Cr_{0.2}$-NH_2(40)	*Cc*	19.651(12)	7.705(03)	6.581(03)	105.08(06)	6.97*
	Imma	6.622(04)	17.256(18)	12.499(10)	90.00	
$Al_{0.8}Fe_{0.2}$-NH_2(100)	*Cc*	19.855(12)	7.701(02)	6.660(02)	105.59(04)	2.52
$Al_{0.8}Fe_{0.2}$-NH_2(80)	*Cc*	19.811(11)	7.691(03)	6.644(02)	105.44(04)	10.37
$Al_{0.8}Fe_{0.2}$-NH_2(60)	*Cc*	19.764(15)	7.693(04)	6.637(03)	105.29(05)	10.05
$Al_{0.8}Fe_{0.2}$-NH_2(40)	*Cc*	19.660(17)	7.655(04)	6.632(03)	105.01(05)	9.69

*The refinement was performed by assuming the simultaneous presence of a *np-phase* (*Cc*) and a *lp*-phase (*Pnma* or *Imma*).

6. Temperature-dependent PXRD patterns of MIL-53($Al_{0.8}Cr_{0.2}$)-NH_2(X) materials

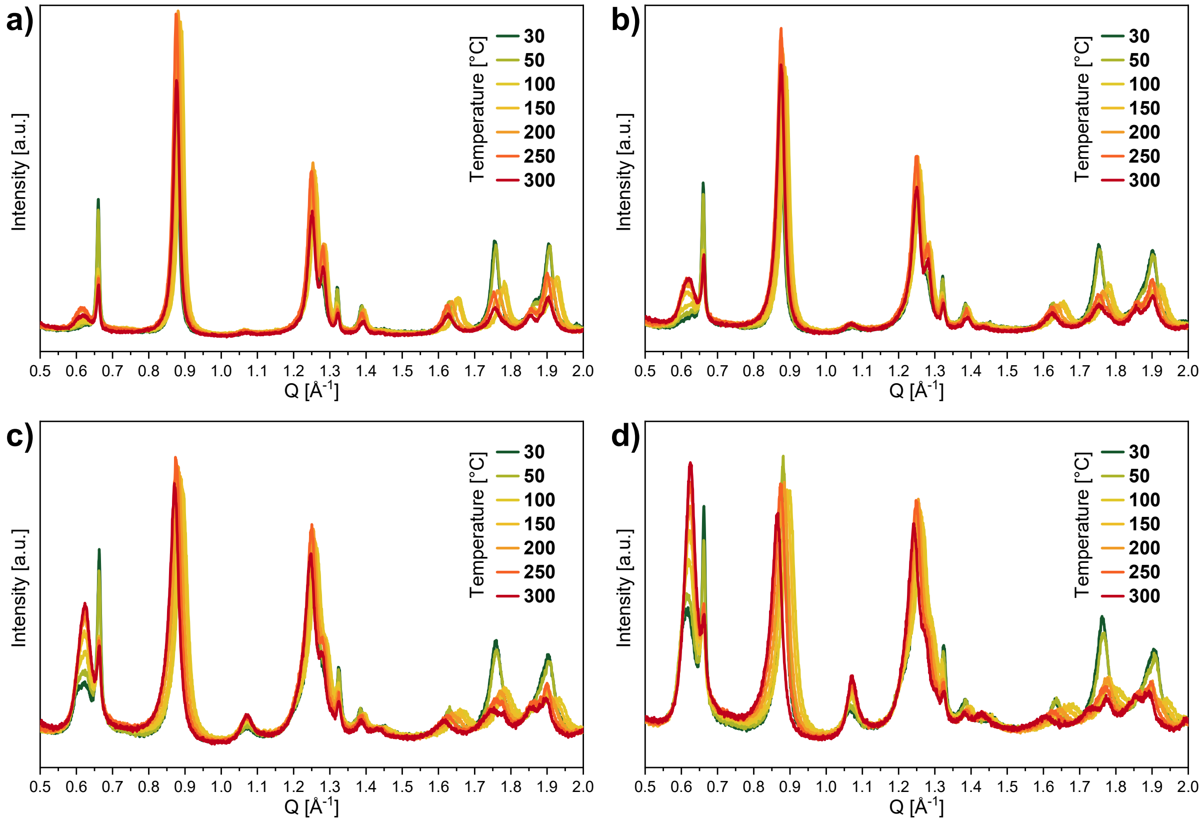

Figure S3. Powder X-ray diffraction patterns of MIL-53($Al_{0.8}Cr_{0.2}$)-NH_2(X) materials recorded *in situ* at different temperatures in nitrogen gas atmosphere at ambient pressure (X = 100 (**a**), 80 (**b**), 60 (**c**) or 40 (**d**)).

7. Post-synthetically modified MIL-53($Al_{0.8}M_{0.2}$)-NH_2(X)-Mal materials

Table S4. Results of the performed unit cell refinements (Pawley method) of post-synthetically modified MIL-53($Al_{0.8}M_{0.2}$)-NH_2-(X)-Mal materials.

	space group	a [Å]	b [Å]	c [Å]	β [°]	R_{wp} [%]
MIL-53($Al_{0.8}Sc_{0.2}$)-NH_2(100)-Mal	*Cc*	19.743(18)	8.107(4)	6.704(4)	106.34(15)	11.33
MIL-53($Al_{0.8}Sc_{0.2}$)-NH_2(80)-Mal	*Cc*	19.575(97)	8.743(4)	6.696(6)	107.27(90)	10.46
MIL-53($Al_{0.8}Sc_{0.2}$)-NH_2(60)-Mal	No conclusive refinement possible					
MIL-53($Al_{0.8}Sc_{0.2}$)-NH_2(40)-Mal	No conclusive refinement possible					
MIL-53($Al_{0.8}V_{0.2}$)-NH_2(100)-Mal	*Cc*	18.626(5)	11.876(3)	6.632(1)	111.07(4)	7.79
MIL-53($Al_{0.8}V_{0.2}$)-NH_2(80)-Mal	*Cc*	18.562(14)	11.968(3)	6.620(1)	111.53(10)	11.70
MIL-53($Al_{0.8}V_{0.2}$)-NH_2(60)-Mal	*Cc*	18.289(9)	12.428(2)	6.642(1)	111.01(06)	7.15
MIL-53($Al_{0.8}V_{0.2}$)-NH_2(40)-Mal	*Cc*	18.222(27)	12.468(3)	6.630(1)	111.12(22)	9.09
MIL-53($Al_{0.8}Cr_{0.2}$)-NH_2(100)-Mal	*Cc*	18.703(10)	11.673(2)	6.622(1)	111.04(7)	8.73
MIL-53($Al_{0.8}Cr_{0.2}$)-NH_2(80)-Mal	*Cc*	18.562(6)	11.913(1)	6.616(1)	111.15(5)	8.24
MIL-53($Al_{0.8}Cr_{0.2}$)-NH_2(60)-Mal	*Cc*	18.656(17)	11.791(6)	6.619(2)	111.30(11)	10.10
MIL-53($Al_{0.8}Cr_{0.2}$)-NH_2(40)-Mal	No conclusive refinement possible					
MIL-53($Al_{0.8}Fe_{0.2}$)-NH_2(100)-Mal	*Cc*	19.800(20)	7.834(5)	6.705(5)	105.39(10)	14.49
MIL-53($Al_{0.8}Fe_{0.2}$)-NH_2(80)-Mal	No conclusive refinement possible					
MIL-53($Al_{0.8}Fe_{0.2}$)-NH_2(60)-Mal	*Cc*	18.753(27)	11.920(9)	6.646(3)	111.57(17)	9.00
MIL-53($Al_{0.8}Fe_{0.2}$)-NH_2(40)-Mal	No conclusive refinement possible					

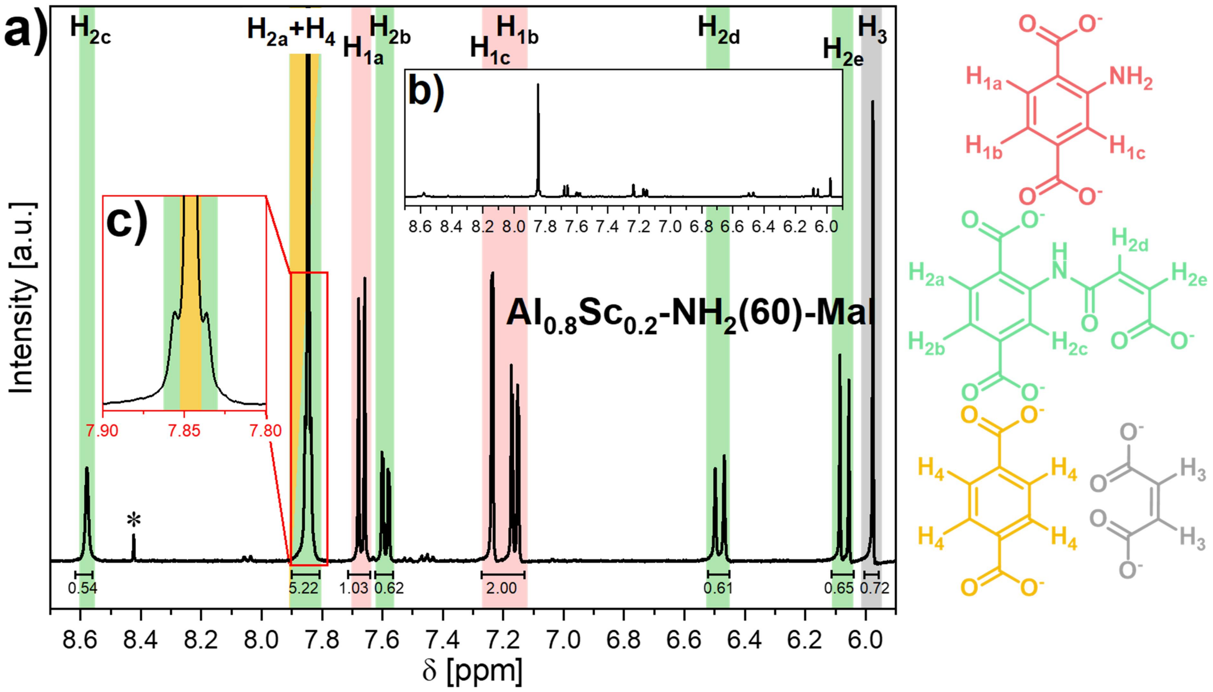

Figure S4. Exemplary ^{1}H-NMR spectrum of post-synthetically modified MIL-53($Al_{0.8}Sc_{0.2}$)-NH_2(60)-Mal, and the corresponding assignment of the protons. This spectrum is representative for all MIL-53($Al_{0.8}M_{0.2}$)-NH_2(X)-Mal materials. The decomposition of DMF during the synthesis resulted in the formation of formate species (*), which could not be removed during the workup. A zoomed-in view (**a**) and an overview (**b**) of the region of aromatic protons. Magnification of the signal centered at 7.85 ppm (**c**).

Table S5. Percentage of post-synthetically modified 2-aminoterephthalate linkers and the overall modification degree with respect to the sum of all linker molecules of MIL-53($Al_{0.8}M_{0.2}$)-NH_2(X)-Mal materials. Micropore volumes and specific surface areas of MIL-53($Al_{0.8}M_{0.2}$)-NH_2(X)-Mal materials were calculated from nitrogen physisorption isotherms by using the t-plot or BET method, respectively.

	**modified amine groups** [%]	**overall modification degree** [%]	**micropore volume** [cm² g^{-1}]	**specific surface area S_{BET}** [m^2 g^{-1}]
MIL-53($Al_{0.8}Sc_{0.2}$)-NH_2(100)-Mal	18	18	< 0.01	30
MIL-53($Al_{0.8}Sc_{0.2}$)-NH_2(80)-Mal	36	29	< 0.01	40
MIL-53($Al_{0.8}Sc_{0.2}$)-NH_2(60)-Mal	61	36	< 0.01	70
MIL-53($Al_{0.8}Sc_{0.2}$)-NH_2(40)-Mal	86	34	0.01	110
MIL-53($Al_{0.8}V_{0.2}$)-NH_2(100)-Mal	50	50	< 0.01	60
MIL-53($Al_{0.8}V_{0.2}$)-NH_2(80)-Mal	29	23	0.04	210
MIL-53($Al_{0.8}V_{0.2}$)-NH_2(60)-Mal	31	18	0.03	200
MIL-53($Al_{0.8}V_{0.2}$)-NH_2(40)-Mal	62	25	0.01	100
MIL-53($Al_{0.8}Cr_{0.2}$)-NH_2(100)-Mal	32	32	< 0.01	70
MIL-53($Al_{0.8}Cr_{0.2}$)-NH_2(80)-Mal	20	16	0.01	150
MIL-53($Al_{0.8}Cr_{0.2}$)-NH_2(60)-Mal	59	36	< 0.01	110
MIL-53($Al_{0.8}Cr_{0.2}$)-NH_2(40)-Mal	56	22	0.05	260
MIL-53($Al_{0.8}Fe_{0.2}$)-NH_2(100)-Mal	16	16	< 0.01	90
MIL-53($Al_{0.8}Fe_{0.2}$)-NH_2(80)-Mal	24	19	< 0.01	80
MIL-53($Al_{0.8}Fe_{0.2}$)-NH_2(60)-Mal	53	32	< 0.01	110
MIL-53($Al_{0.8}Fe_{0.2}$)-NH_2(40)-Mal	43	17	0.02	180

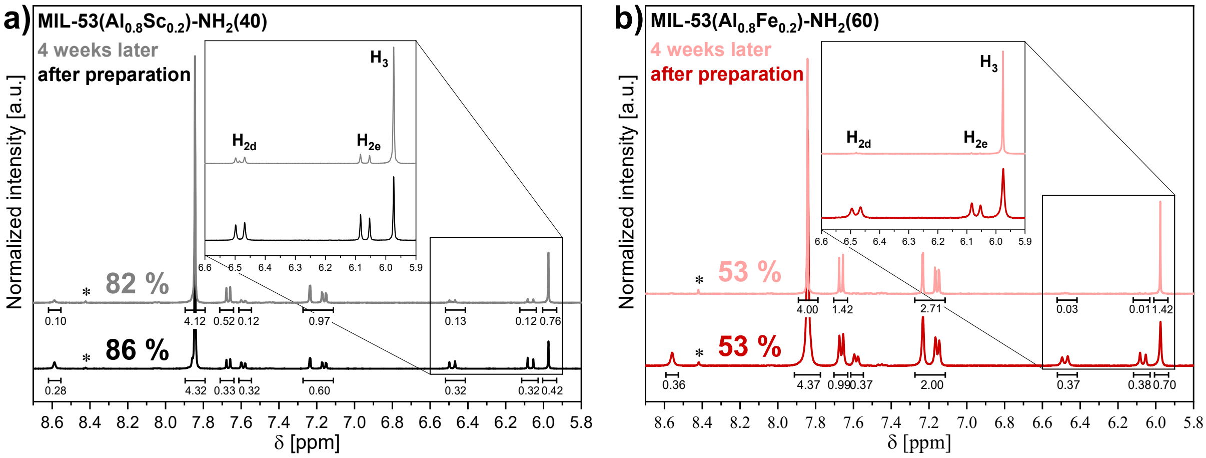

Figure S5. Comparison of ^{1}H-NMR spectra of MIL-53($Al_{0.8}Sc_{0.2}$)-NH_2(40) (**a**) and MIL-53($Al_{0.8}Fe_{0.2}$)-NH_2(60) (**b**) directly after preparation of the measurement solution (dark colors) and four weeks later (light colors). The percentage numbers represent the calculated modification degrees based on Equation (1) in the main manuscript (Experimental Section). The assignment of the signals refers to Figure S4.

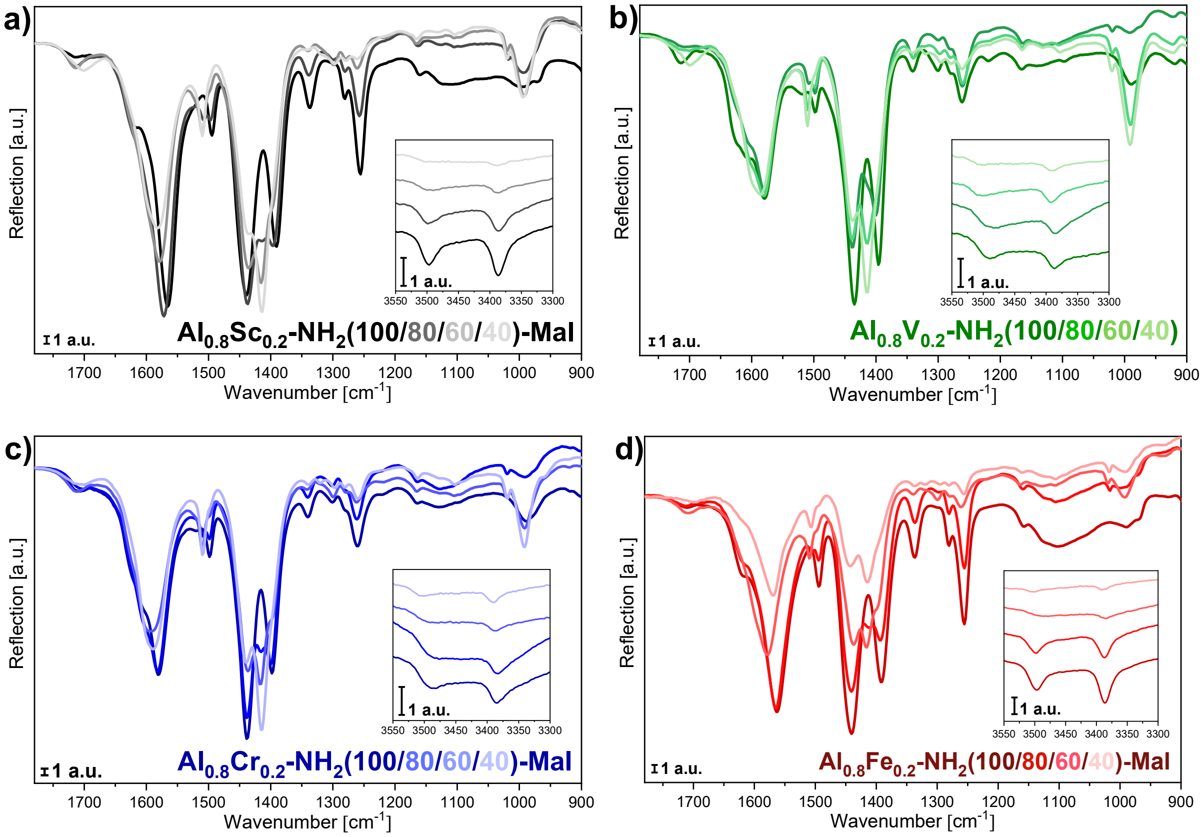

Figure S6. ATR-IR spectra of post-synthetically modified MIL-53($Al_{0.8}M_{0.2}$)-NH_2(X)-Mal materials (X = 100, 80, 60, 40). Sc (**a**), V (**b**), Cr (**c**), Fe (**d**). The insets provide zoomed-in views on the bands of the amine vibrations.
